# Intelligent incremental classification using a dynamic grasshopper-enhanced neural network for data streams

**DOI:** 10.1038/s41598-026-38571-y

**Published:** 2026-02-26

**Authors:** Saad M. Darwish, Noha A. El-Shoafy

**Affiliations:** 1 Department of Information Technology, Institute of Graduate Studies and Research, Alexandria University, 21526 Alexandria, Egypt; 2 Computer Science Department, Faculty of Computers and Artificial Intelligence, Matrouh University, Matrouh, 51511 Egypt

**Keywords:** Dynamic grasshopper optimization, Incremental learning, Intelligent systems, Big data, Neural network optimization, Engineering, Mathematics and computing

## Abstract

Complex data streams are highly dynamic, large-scale, and prone to continuous distributional shifts, posing significant challenges for neural network-based classification systems—particularly in maintaining accuracy and efficiency without frequent retraining. To address these issues, this study proposes an intelligent incremental learning framework that integrates a Dynamic Grasshopper Optimization Algorithm (DGOA) with a Multilayer Perceptron (MLP) neural network for real-time hyperparameter optimization. The proposed system functions as an adaptive intelligent system, utilizing DGOA—an enhanced form of the traditional Grasshopper Optimization Algorithm equipped with dynamic parameter control and online swarm reconfiguration—to autonomously adjust to evolving data characteristics. Unlike conventional GOA, the dynamic variant modifies its search behavior and population dynamics in real time, enabling continuous learning without restarting the optimization process. Through this mechanism, the model incrementally tunes critical hyperparameters such as the learning rate and momentum, resulting in improved accuracy and generalization on unseen data. The main contribution of this research lies in developing a fully online, swarm-intelligence-driven hyperparameter optimization strategy tailored for big data streams. Experimental evaluations on the Australian electricity market dataset demonstrate that the DGOA-based MLP achieves a classification accuracy of 89.5%, outperforming Grid Search (84.2%), Random Search (83.5%), PSO (86.7%), GA (87.1%), ACO (86.9%), and standard GOA (87.8%). Additionally, DGOA reduces the average computational time to 120 s and converges in only 30 iterations while achieving the lowest final loss (0.21), highlighting its superior efficiency and convergence stability.

**Supplementary Information:**

The online version contains supplementary material available at 10.1038/s41598-026-38571-y.

## Introduction

Industrial processes generate massive volumes of data in real time from sensors, equipment, and various connected systems. This data often contains valuable insights that help optimize operations, improve efficiency, and prevent downtime. Streaming technologies enable the ingestion, processing, and analysis of such data in near real time, allowing for rapid responses to changing conditions and operational events^1,2^.

However, handling industrial big data streams presents several unique challenges. The continuous and high-velocity nature of streaming data, coupled with the large scale of industrial systems, requires scalable and high-performance analytics platforms capable of maintaining low latency and high throughput. Moreover, in dynamic industrial environments, data distributions may evolve over time, leading to model drift and a degradation of predictive performance. Therefore, implementing techniques for model monitoring, adaptation, and retraining is crucial to ensuring that predictive models remain accurate and relevant over time^3–5^.

**Fig. 1:**
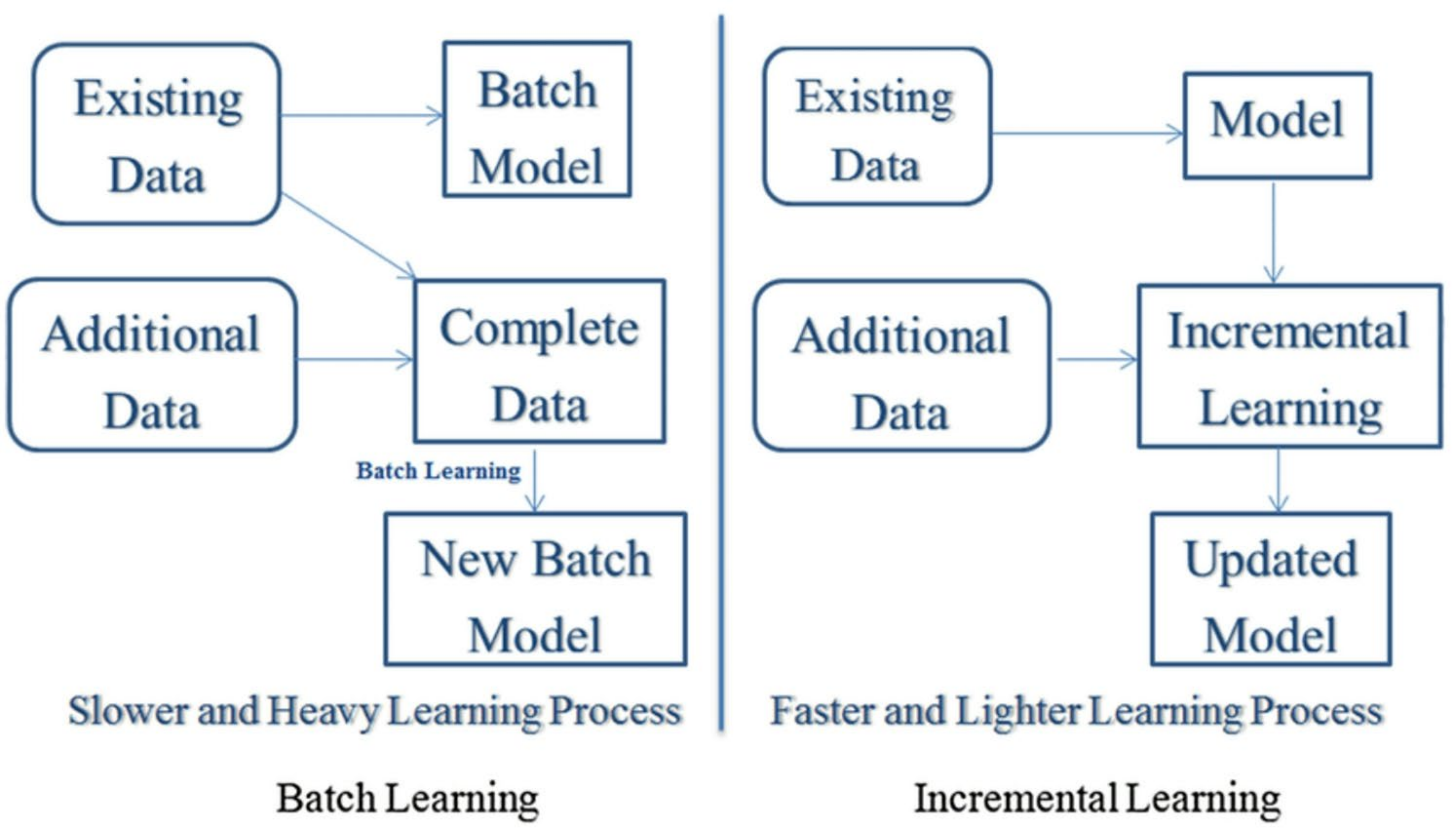
Batch learning versus incremental learning.

Traditional machine learning algorithms face challenges in efficiently handling such big data streams. Many conventional approaches require loading entire datasets into memory, which is infeasible for large-scale streaming data. Batch processing also becomes computationally intensive and time-consuming (see Fig. 1). Furthermore, traditional models are typically trained offline using static historical data and often fail to adapt to evolving data without complete retraining. This lack of adaptability leads to suboptimal performance in dynamic environments. Another concern is that complex deep learning models can be difficult to interpret and debug in real-time scenarios, highlighting the need to balance model complexity and interpretability for reliable decision-making systems.

To overcome these limitations, researchers have developed specialized approaches for handling big data streams, including optimized Artificial Neural Networks (ANNs) designed for real-time operation. These models can efficiently process large volumes of data, adapt to changing distributions, and minimize resource consumption^6,7^, . Optimizing ANNs for streaming contexts involves tuning critical hyperparameters to ensure efficient model training and inference. Automated hyperparameter optimization methods—such as grid search, random search, Bayesian optimization, and evolutionary algorithms—have been widely employed to systematically explore the hyperparameter space and identify the optimal configuration of ANN models^8,9^.

Despite the progress made in traditional and swarm-based optimization algorithms, several limitations hinder their performance in real-time streaming and industrial big data environments. Conventional optimization techniques—such as Grid Search, Random Search, and Bayesian Optimization—are computationally expensive and often unsuitable for continuous data streams, as they require retraining models from scratch each time new data arrives^10–12^.

Similarly, while evolutionary and swarm intelligence algorithms—such as Genetic Algorithms (GA), Particle Swarm Optimization (PSO), and the standard GOA—have demonstrated strong global search capabilities, they are generally designed for static datasets and offline optimization. These methods typically assume fixed data distributions and may fail to adapt dynamically as the underlying data characteristics evolve, leading to premature convergence or suboptimal parameter tuning^13,14^.

Moreover, in high-velocity industrial data streams, latency and resource constraints make frequent retraining or large-scale parameter searches impractical. The standard GOA, though effective at balancing exploration and exploitation, relies on static control parameters as the comfort zone coefficient ($\:c$) and social interaction coefficient ($\:f$) which limits its responsiveness to evolving data patterns. This restricts its use in incremental learning scenarios, where real-time hyperparameter adjustment is critical to maintaining model accuracy and stability^13^.

To address these drawbacks, the present work introduces DGOA integrated with an incremental MLP framework. The proposed DGOA adaptively updates its control parameters in real time, allowing it to respond to data drift and streaming variability. By dynamically tuning the MLP’s learning rate and momentum, the method achieves faster convergence, a lower computational cost, and continuous adaptation without retraining from scratch. A detailed comparison between the traditional GOA and the proposed DGOA in the context of real-time data stream optimization is presented in Table 1.

The superior performance of the proposed DGOA over the original GOA can be attributed to its adaptive dynamic control strategy and enhanced response to non-stationary data environments. In traditional GOA, the attraction–repulsion forces among agents are governed by fixed coefficients, which may cause premature convergence or stagnation when the search space or data distribution evolve over time.

DGOA introduces time-varying control parameters that adjust dynamically based on feedback from the optimization process—such as convergence rate, error variance, and population diversity. This adaptation enables DGOA to maintain a balanced trade-off between global exploration and local exploitation throughout the optimization process. Moreover, DGOA integrates a data drift detection mechanism that modifies the search intensity when changes in the data stream are detected, preventing the optimizer from becoming biased toward outdated patterns. As a result, DGOA converges more quickly to the optimal hyperparameters for the neural network while avoiding oscillations and local minim.

**Table 1:** Comparison between traditional GOA and dynamic GOA (DGOA) for Real-Time data Streaming.

Feature	Traditional GOA	Dynamic GOA (DGOA)
Optimization Type	Static/Offline	Dynamic/Online (Real-time)
Control Parameters	Fixed attraction and comfort coefficients	Adaptively updated coefficients based on data stream characteristics
Exploration–Exploitation Balance	Gradually shifts using a predefined decay rate	Continuously adjusted using real-time feedback from convergence and data drift metrics
Responsiveness to Data Drift	Low — requires re-initialization or retraining	High — automatically adapts without retraining
Computational Overhead	Higher for repeated optimization runs	Lower — supports continuous incremental updates
Suitability for Streaming Environments	Limited (static data only)	Excellent (real-time, non-stationary data)
Integration with Incremental MLP	Manual tuning of hyperparameters	Automated dynamic tuning (learning rate, momentum)

### Problem statement

In industrial environments such as manufacturing plants or utility facilities, massive volumes of data are continuously generated by various sensors, equipment, and control systems. These industrial big data streams contain valuable insights that, if processed and analyzed in real time, can significantly enhance efficiency, productivity, and enable predictive maintenance.

However, traditional data processing methods are often inadequate for managing the volume, velocity, and variety of such data streams. These limitations hinder the ability to extract timely and actionable insights that are necessary for informed decision-making. Therefore, the main challenge is to develop an intelligent system capable of processing industrial big data streams in real time, extracting useful patterns, and enabling dynamic decision-making to optimize operational efficiency, enhance quality control, and reduce downtime.

To achieve this, models must be adaptive and capable of learning from continuously arriving data without experiencing performance degradation. Incremental learning offers a viable solution by enabling models to update and refine their knowledge as new data become available while maintaining stability and reliability.

### Motivation of the work

Industrial big data streams originate from dynamic and rapidly changing environments. Incremental learning techniques are well-suited for such scenarios because they allow models to adapt in real time to evolving data distributions and trends. This adaptability ensures that predictions remain accurate and relevant over time.

However, applying incremental learning to ANNs introduces several challenges. Incremental training can lead to instability, oscillations, or even divergence in the learning process. Ensuring convergence requires careful tuning of hyperparameters such as the learning rate, regularization strength, and optimization strategy. Thus, hyperparameter optimization becomes essential to improving ANN performance, generalization ability, and robustness in handling continuous data streams.

To address these challenges, the proposed system employs a DGOA designed to adapt dynamically to incoming data streams. The DGOA continuously updates its optimization process in real time as new data arrive, optimizing key ANN hyperparameters. Fine-tuning these parameters enhances model accuracy and generalization on unseen data. Moreover, the DGOA demonstrates fast convergence and efficiently identifies high-quality solutions within a small number of iterations, making it suitable for time-sensitive and resource-constrained industrial applications.

### Contribution and novelty

The primary contribution of this research is the development of a fully online, dynamic hyperparameter optimization framework for incremental neural network learning in non-stationary big data stream environments. Unlike existing neural network–metaheuristic hybrid approaches, which typically perform hyperparameter optimization in an offline or batch-oriented manner, the proposed method fundamentally differs by enabling continuous, real-time optimization without restarting the learning or optimization process.

The novelty of the proposed approach lies in the integration of a DGOA with an incremental MLP. In contrast to traditional GOA-based or other swarm intelligence hybrids that rely on fixed control parameters and static fitness landscapes, DGOA introduces adaptive, time-varying control parameters that evolve based on real-time feedback from the learning process, including convergence behavior and performance variation due to data drift.

Key novel aspects of this work include:

1. Online hyperparameter optimization: Hyperparameters such as learning rate and momentum are continuously adjusted during incremental learning, eliminating the need for retraining or repeated optimization cycles.
2. Dynamic swarm adaptation: The optimization process adapts its exploration–exploitation balance in response to changing data distributions, making it suitable for non-stationary streaming environments.
3. Drift-aware incremental learning: The proposed framework maintains hyperparameter relevance and model stability under concept drift, which is not addressed by conventional NN–metaheuristic hybrids.
4. Computational efficiency: By avoiding repeated reinitialization and batch retraining, the proposed method significantly reduces computational overhead, making it practical for real-time industrial applications.

Overall, this work advances the state of the art by transforming swarm intelligence–based neural network optimization from a static, offline process into a dynamic, adaptive, and fully incremental learning paradigm, specifically designed for real-time big data stream classification.

 The remainder of this article is organized as follows: a literature review on hyperparameter optimization methods is presented in Sect. 2. The proposed strategy is detailed in Sect. 3. Section 4 presents the evaluation, results, and discussion. Finally, Sect. 5 provides a concise summary of the study and offers suggestions for future research.

## Related work

Incremental classification techniques play a crucial role in managing big data efficiently. By continuously updating models with new data, these techniques ensure that the models remain accurate and relevant over time. Generally, current incremental classification techniques in the context of big data can be categorized into five main groups^15–17^.

- Online learning algorithms, such as stochastic gradient descent (SGD), update model weights incrementally for each new data point or batch, minimizing the loss function.
- Ensemble methods combine multiple models to improve robustness, where new data can either update existing models or introduce new ones into the ensemble.
- Incremental clustering methods, such as incremental k-means, update cluster centroids as new data arrives.
- Neural networks using incremental backpropagation update network weights incrementally with incoming samples.
- Probabilistic models, such as Hidden Markov Models (HMMs), can be updated incrementally for time-series data.

Each approach has distinct strengths and suitable application areas. Factors such as scalability, concept drift, memory management, and model complexity must be carefully considered when selecting an appropriate technique^18,19^.

### Hyperparameter optimization in incremental learning

Optimizing hyperparameters for neural networks, especially in incremental classification, is a critical factor influencing performance. Incremental learning requires models to learn continuously without forgetting previously acquired knowledge, demanding adaptive and efficient optimization strategies.

Existing studies on hyperparameter optimization can be broadly grouped into heuristic-based, swarm intelligence–based, and evolutionary or reinforcement learning–based methods. The following subsections discuss these categories while highlighting their main limitations in dynamic environments.

#### Heuristic-based and gradient-free optimization methods

In Ref.^9^, the authors proposed an Adaptive Teaching Learning-Based (ATLB) heuristic to identify optimal hyperparameters across various network architectures. Similarly, Ref.^20^ introduced an algorithm based on simplified swarm optimization for hyperparameter optimization in convolutional neural networks (CNNs). While these methods reduce computational overhead, they are often designed for static datasets and require retraining when data distributions shift, making them less effective in incremental settings.

#### Reinforcement learning and evolutionary optimization

Further advancements include reinforcement learning–based optimization. In Ref.^21^, researchers employed Q-learning for hyperparameter optimization, where an agent continuously updates its Q-table to improve performance. Their modified Q-learning approach, with adaptive starting states and termination criteria, achieved better performance than Tree of Parzen Estimators (TPE), random search, and GA on both bidirectional LSTM and CNN models.

Evolutionary algorithms have also been applied in various domains. Reference^11^ proposed an evolutionary approach for optimizing hyperparameters in pre-trained CNN models for insect pest classification. A population-based optimization approach, HyperBRKGA, was proposed in Ref.^22^ to achieve faster and more accurate hyperparameter tuning. Although these methods effectively explore large search spaces, they typically require high computational cost and lack real-time adaptability to new data, which limits their applicability in continuous learning environments.

#### Swarm intelligence–based optimization

In Ref.^23^, the Arithmetic Optimization Algorithm (AOA) was introduced as a metaheuristic for tuning deep neural network (DNN) hyperparameters. In Ref.^24^, the Salp Swarm Algorithm (SSA) achieved 99.46% classification accuracy on the MNIST dataset, and Ref.^25^ employed the Binary Bat Algorithm (BBA) for handwritten Chinese character classification. While swarm-based techniques often achieve impressive accuracy, they are prone to premature convergence and may not generalize well under concept drift scenarios.

#### Differential evolution and hybrid frameworks

A universal framework, DE-Opt, was proposed in Ref.^26^ for deep learning–based recommender systems, integrating Differential Evolution (DE) for layer-wise learning rate and regularization tuning. Similarly, Refs.^27,28^, explored PSO, GA, and SSO for CNN optimization in various domains, while Ref.^29^ developed a Dynamic Bayesian hyperparameter optimization approach for resource-efficient cloud-based networks. Despite their strong optimization capabilities, most of these approaches do not support incremental updating, meaning their optimized parameters become suboptimal as new data arrives.

### Incremental learning with adaptive optimization

For incremental learning in data streams, Ref.^30^ proposed an optimization module that dynamically updates neural network parameters using a loss-based concept drift prediction function. Similarly, Ref.^31^ introduced a dynamic clustering approach that accounts for concept drift using supervised clustering and automated group elimination.

In Ref.^32^, the Incremental Adaptive and Heterogeneous Ensemble (IAHE) model was developed for credit scoring, capable of handling drifting variables and streaming big data efficiently. Reference^33^ further enhanced this direction by introducing a cluster-based active learning approach to manage class imbalance and concept drift. While these methods demonstrate adaptability in incremental learning, they often lack integrated hyperparameter optimization mechanisms, which limits their ability to maintain long-term performance stability in evolving data streams.

Recent studies have extended incremental learning techniques to enhance the adaptability of deep neural networks in evolving data streams. For instance, Zhong et al.^34^ proposed a Robust Incremental Broad Learning System capable of handling data streams of uncertain scale without full retraining, maintaining high predictive accuracy. Similarly, Moradi et al.^35^ employed unsupervised domain adaptation to adjust models dynamically for concept-drifting data streams. Earlier work by Ashfahani^36^ introduced autonomous deep learning, incrementally updating DNN structures to continuously incorporate new data, while Li et al.^37^ demonstrated memory-based estimation and event-driven control for distributed networked systems.

In contrast, the proposed approach combines incremental classification with adaptive hyperparameter optimization, ensuring both continual learning and efficient parameter tuning. This integration addresses the computational inefficiency and lack of adaptability observed in prior work. For a more comprehensive review of incremental learning and neural network hyperparameter optimization techniques, readers may refer to Ref.s^38–42^.

### Metaheuristic optimization: a review of recent advances

Recent research has increasingly focused on employing metaheuristic optimization algorithms to address complex problems in diverse computational domains. In Ref.^40^, the authors explored the application of these algorithms for detecting textual cyber harassment, systematically evaluating their role in improving text classification accuracy.

Similarly, Abualigah et al.^41^ applied gradient-based optimization in software engineering, achieving notable efficiency gains. Building on these efforts, AlShorman et al.^42^ conducted a systematic review of machine learning techniques for smart contract security, identifying key challenges in blockchain environments. Furthermore, the authors in Ref.^43^ advanced pattern recognition through a neural network framework for handwritten Arabic recognition, demonstrating superior accuracy using innovative feature extraction techniques.

A significant portion of recent studies have focused on nature-inspired and hybrid metaheuristic models to enhance optimization performance across engineering and computational problems. For example, Abu-Hashem^44^improved the Black Widow Optimization (BWO) algorithm for efficient cloud task scheduling, while the work presented in Ref.^45^. provided a comprehensive survey of BWO variants and their diverse applications.

Similarly, Almodfer et al.^46^proposed a hybrid model that integrates the Reptile Search Algorithm with local search to improve convergence and solution precision. In a broader context, the authors in Ref.^47^ reviewed multiple classes of metaheuristics, highlighting ongoing challenges in achieving a balance between exploration and exploitation. Furthermore, the work in Ref.^48^ introduced an enhanced hybrid metaheuristic tailored for engineering design optimization, demonstrating superior convergence compared to conventional algorithms.

Beyond optimization, researchers have begun to integrate these computational techniques into applied intelligent systems. For instance, Abualigah et al.^49^ reviewed the Krill Herd Algorithm, offering valuable insights into its biological foundations and practical applications. In a different domain, Shehab^50^ developed a simulation-driven free-fall model for generating synthetic data to train deep learning systems in disaster management, effectively addressing the challenge of data scarcity.

Furthermore, AbuAladas et al.^51^ combined the Scatter Search Algorithm with Support Vector Machines for intrusion detection in IoT networks, significantly improving detection accuracy and reducing false alarms. Collectively, these studies demonstrate the growing importance of hybrid metaheuristics and intelligent optimization techniques in solving real-world engineering, security, and data-driven challenges.

Recent years have seen the growing integration of nature-inspired optimization with deep learning and time-series forecasting to enhance model performance, convergence, and generalization. For example, in Ref.^52^, the authors introduced an Improved Sand Cat Swarm Optimization (ISCSO) algorithm to tune the GraphSAGE-GRU network for predicting the remaining useful life (RUL) of engines, achieving significant gains in accuracy and stability.

Similarly, Yuan et al.^53^ proposed a Combined Improved Tuna Swarm Optimization (ITSO) algorithm integrated with a Graph Convolutional Neural Network (GCN), enabling effective spatio-temporal feature learning for RUL estimation. Together, these studies demonstrate the power of metaheuristic-assisted learning frameworks, though they are primarily designed for static or offline datasets in which full retraining is feasible after data updates.

In parallel, optimization-enhanced approaches have also been applied to forecasting and engineering system design. For instance, in Ref.^54^, the authors developed a Short-Term Power Load Forecasting Model (SKDR) that combines a stacked ensemble with Sparrow Search Optimization to fine-tune model parameters, achieving high predictive accuracy for static load data.

Meanwhile, the work presented in Ref.^55^ proposed a Multidisciplinary Design Optimization (MDO) framework for the dynamic positioning system of a semi-submersible platform, integrating mechanical, control, and hydrodynamic parameters into a unified optimization process.

Although these studies demonstrate the versatility of optimization in various domains, they remain limited to offline or deterministic contexts. In contrast, our proposed DGOA extends swarm intelligence to real-time, streaming environments, continuously adapting neural network hyperparameters without full retraining—offering a novel and scalable solution for industrial big data analytic.

While previous studies^40–51^ have demonstrated the effectiveness of various metaheuristic algorithms in enhancing convergence and problem-solving efficiency, these approaches still face persistent challenges. Common challenges include premature convergence, local optima entrapment, and an imbalanced trade-off between exploration and exploitation, particularly in high-dimensional or dynamic environments.

In contrast, the proposed DGOA eliminates the static data assumption by integrating online adaptation mechanisms into its swarm dynamics. Through dynamic step-size adjustment and adaptive attraction coefficients, DGOA maintains a continuous balance between exploration and exploitation as new data arrive.

This adaptive design enables real-time parameter tuning without full retraining, directly addressing the limitations of earlier metaheuristic methods and positioning DGOA as a robust optimization framework for non-stationary, streaming, and evolving problem spaces.

### Research gap

Incremental classification techniques are essential for managing large-scale and continuously evolving data. However, most existing methods still face limitations in effectively addressing concept drift and maintaining performance in high-dimensional or streaming environments. Moreover, many rely on fixed hyperparameters, restricting their adaptability to evolving data characteristics and leading to performance degradation over time.

Optimizing neural network hyperparameters in incremental learning presents additional challenges. As new data arrives, shifts in the underlying distribution can render previously tuned hyperparameters suboptimal. Ensuring scalability and computational efficiency in this optimization process is also critical, as traditional methods—such as grid search or evolutionary algorithms—often require restarting the tuning process with each data update, which is unsuitable for real-time applications.

To overcome these challenges, this research proposes an adaptive incremental learning framework that integrates dynamic hyperparameter optimization. The system employs adaptive algorithms capable of adjusting network parameters on the fly as new data is processed, maintaining responsiveness to evolving patterns without retraining from scratch.

Specifically, while the traditional GOA exhibits limited adaptability in streaming contexts, the proposed DGOA incrementally updates solutions as new data becomes available. By continuously refining the optimization process, DGOA maintains parameter relevance and model accuracy while effectively detecting and adapting to concept drift. This ensures consistent predictive performance in dynamic industrial data environments^56–58^.

## Methodology

This section presents the proposed model for optimizing the hyperparameters of an ANN using the DGOA. The primary objective is to achieve the best possible incremental classification accuracy and processing speed for big data stream applications. The proposed model employs a train-and-forget strategy, specifically designed to handle the continuous and infinite nature of big data streams. In this strategy, the model processes and learns from only a portion of the incoming data stream at a time—without retaining or revisiting historical data.

**Fig. 2:**
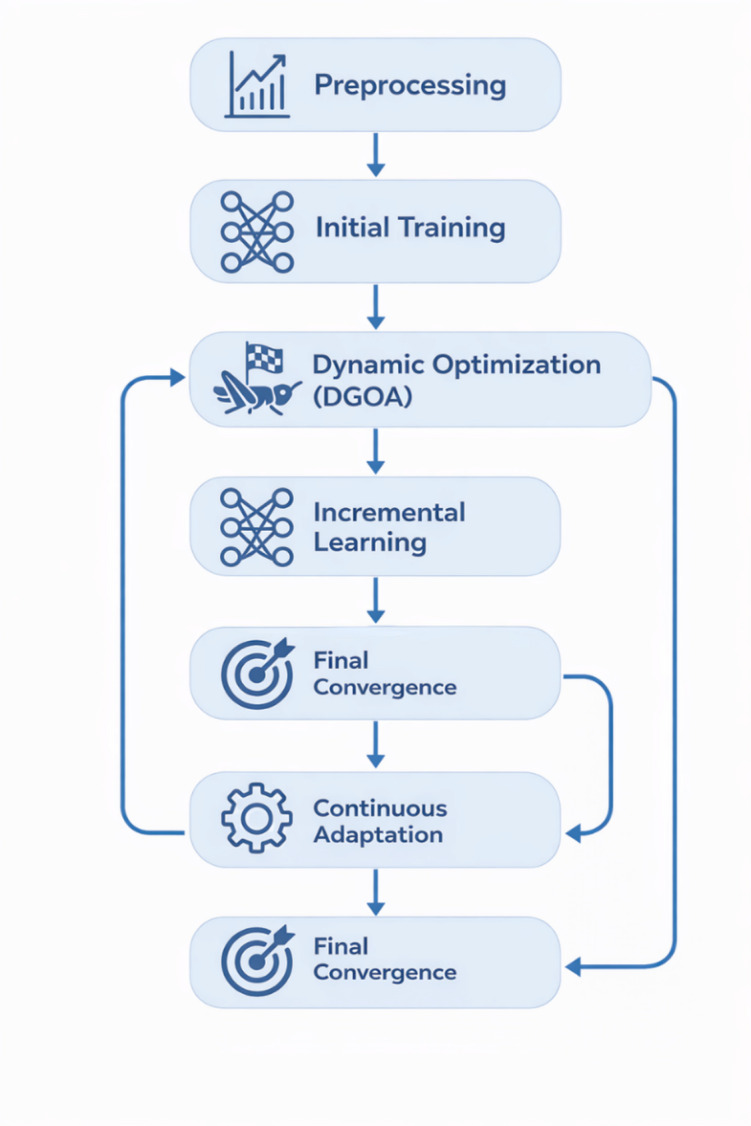
Process flow diagram of the proposed DGOA model.

This design allows for efficient operation in memory-constrained environments while dynamically adapting to evolving data distributions. Figure 2 provides a schematic overview of the proposed DGOA, detailing the initialization, dynamic parameter adjustment, fitness evaluation, and convergence processes that guide hyperparameter optimization in the MLP model.

Each new segment of the data stream triggers an incremental update to the model’s internal parameters, such as weights and biases, ensuring that the model remains aligned with the latest data trends. By fine-tuning existing weights during each update, the model reduces the computational overhead associated with retraining from scratch.

Consequently, the train-and-forget approach supports real-time learning and decision-making, enabling the continuous integration of new information while discarding outdated knowledge. This ensures scalability and makes the model well-suited for scenarios in which data streams may grow indefinitely over time.

When a new segment arrives, it is treated as a fresh training instance, prompting the model to incrementally update the MLP classifier. This process ensures that the classifier reflects the most recent data patterns and relationships, maintaining high adaptability and accuracy over time^59^. The model begins by training on the initial segment of the data stream to establish baseline hyperparameter values for the MLP neural network—specifically the learning rate ($\:\eta\:$) and momentum ($\:\mu\:$).

The learning rate ($\:\eta\:$) controls the magnitude of weight updates during gradient descent. Smaller $\:\eta\:$ values lead to precise but slower convergence, whereas larger values accelerate learning but risk overshooting the optimal solution.

The momentum parameter ($\:\mu\:$) introduces a smoothing effect by incorporating a fraction of the previous update step, enabling the model to traverse shallow regions of the loss surface efficiently and avoid local minima^10,60^.

As new data segments arrive, the model performs a dynamic hyperparameter optimization process to adapt to the changing data distribution. This iterative adjustment continuously seeks the optimal combination of $\:\eta\:\:$and $\:\mu\:$ to balance learning speed and stability.

By fine-tuning these hyperparameters, the MLP maintains high classification accuracy while minimizing the risk of overfitting or underfitting. This adaptive mechanism ensures that the classifier remains consistently tuned as the data evolves, achieving stable performance across diverse and rapidly changing streaming environments.

The proposed model utilizes the DGOA to automatically adapt the MLP’ hyperparameters in real time. The dynamic adaptation capability of DGOA allows the optimization process to remain responsive to changes in the data stream. Unlike the traditional GOA, DGOA introduces adaptive components that adjust key parameters—such as step size, exploration–exploitation balance, and search space constraints—as the optimization progresses. These modifications enable DGOA to enhance convergence speed and solution quality, particularly in complex or rapidly changing environments such as big data streams^61^.

In the proposed model, a dynamic step size is applied to ensure more flexible navigation within the search space. This approach allows the optimizer to refine its search granularity based on the real-time behavior of the objective function. To summarize, the optimization process using DGOA for tuning η and µ of the MLP neural network can be structured as a series of adaptive steps, where the algorithm continuously learns from streaming data to refine its hyperparameter settings. This integration of DGOA with incremental learning ensures fast convergence, adaptability, and robust classification performance in dynamic big data environments.

### Step 1: data stream preprocessing

The classification of big data streams using a MLP involves real-time processing and decision-making on continuous, high-velocity data inputs. The goal is to design an efficient, incremental learning model that can classify the incoming data into predefined categories while addressing the unique challenges posed by the dynamic nature of data streams. The data stream is characterized by its high volume, velocity, and variability. Traditional batch training is impractical for data streams. Preprocessing is applied to ensure compatibility with incremental learning:

*Data normalization* Scale the features of the input data $\:x$ to a standard range, e.g., [0,1], using:

1 $$\:\acute{x}=\frac{x-\mathrm{m}\mathrm{i}\mathrm{n}\left(x\right)}{\mathrm{max}\left(x\right)-\mathrm{m}\mathrm{i}\mathrm{n}\left(x\right)}$$

*Windowing* The data stream is divided into manageable chunks of size w, where w represents the window size. Selecting the optimal window size for processing data streams involves balancing latency, accuracy, and computational efficiency.

A smaller window size allows the model to respond quickly to changes in the data distribution, making it suitable for dynamic environments. However, it may provide insufficient data for meaningful learning and can increase variability in model updates.

Conversely, a larger window size offers more data for stable learning, but it increases processing delays and risks overlooking short-term patterns or trends. Therefore, the ideal window size depends on the specific characteristics of the data stream, such as its velocity, variability, and the nature of the classification task.

Empirical methods—such as cross-validation or experimentation with different window sizes on historical data—can help identify the configuration that achieves the optimal trade-off between accuracy and processing time. Additionally, adaptive windowing, where the window size $\:w$ is dynamically adjusted based on real-time data trends, can be an effective approach in highly variable or non-stationary data environments.

#### Step 2: initial training on the first data segment

To initialize the MLP model and establish baseline hyperparameters $\:\eta\:$ and $\:\mu\:$ for the first segment of the data stream. Train the MLP classifier on the first data segment to set initial values for the learning rate and momentum.

2 $$\:{w}_{i}\left(t+1\right)={w}_{i}\left(t\right)-\eta\:\left(t\right)\times\:\nabla\:L\left(w\left(t\right)\right)$$

3 $$\:\varDelta\:\mathrm{w}\left(t+1\right)=\mu\:\left(t\right)\times\:\varDelta\:w\left(t\right)-\eta\:\left(t\right)\times\:\nabla\:L\left(w\left(t\right)\right)$$

Where $\:{w}_{i}\left(t\right)$ is the weight at iteration$\:\:t$, $\:\eta\:\left(t\right)$ is the learning rate, and $\:\nabla\:L\left(w\left(t\right)\right)$ is the gradient of the loss function with respect to the weight at time $\:t$, $\:\mu\:\left(t\right)$ is the momentum parameter and $\:\varDelta\:\mathrm{w}\left(t\right)$ is the change in weights at time $\:t$.

In general, using a MLP model for big data streaming classification offers several advantages. MLPs excel at learning complex, non-linear relationships within data, enabling them to handle diverse and high-dimensional input features effectively. Their flexibility allows for incremental updates, making them suitable for real-time streaming environments where data is dynamic and continuously evolving. Additionally, MLPs are model-agnostic to the type of input data, seamlessly integrating numerical, categorical, or mixed data types. When optimized with appropriate techniques, such as adaptive learning and hyperparameter tuning, MLPs can achieve high classification accuracy and adaptability while maintaining computational efficiency, making them a robust choice for processing and analyzing big data streams^10,60^.

#### Step 3: dynamic hyperparameter optimization using DGOA

To optimize the hyperparameters dynamically based on the current segment of data, adjusting the learning rate $\:\eta\:$ and momentum $\:\mu\:$ for improved convergence and accuracy. We apply the dynamic step size in the DGOA to adaptively optimize $\:\eta\:$ and $\:\mu\:$ after each new data segment.

4 $$\:\alpha\:\left(t\right)={\alpha\:}_{max}(1-\frac{t}{T})$$

where $\:{\alpha\:}_{max}$ is the maximum step size, and $\:T$ is the total number of iterations. This causes the step size to decrease as the algorithm progresses, focusing on fine-tuning near the optimal solution. Furthermore, we adjust the exploration factor based on the progress:

5 $$\:\beta\:\left(t\right)={\beta\:}_{initial}(1-\frac{t}{T})$$

where $\:{\beta\:}_{initial}$​ is the initial exploration parameter, decreasing as the optimization process moves toward exploitation. Based on the dynamic step size and exploration factor, adjust the learning rate and momentum:

6 $$\:\eta\:\left(t+1\right)=\eta\:\left(t\right)+\alpha\:\left(t\right)\times\:\delta\:$$

7 $$\:\mu\:\left(t+1\right)=\mu\:\left(t\right)+\alpha\:\left(t\right)\times\:\delta\:$$

where $\:\eta\:\left(t\right)$ and $\:\mu\:\left(t\right)$ are updated using the dynamic parameters.$\:\:\delta\:$ represents the adjustment needed to move closer to the optimal solution.

#### Step 4: incremental training with dynamic hyperparameters

After optimizing the hyperparameters, the model trains incrementally on the next data segment, updating the weights and biases based on the new optimal hyperparameter values $\:\eta\:\left(t+1\right)$ and $\:\mu\:\left(t+1\right)$. In this case, the MLP model is updated incrementally without recalling past segments, ensuring that only the most recent data influences the weights and hyperparameters.

8 $$\:{w}_{i}\left(t+1\right)={w}_{i}\left(t\right)-\eta\:\left(t+1\right)\times\:\nabla\:L\left(w\left(t\right)\right)$$

9 $$\:\varDelta\:\mathrm{w}\left(t+1\right)=\mu\:\left(t+1\right)\times\:\varDelta\:w\left(t\right)-\eta\:\left(t+1\right)\times\:\nabla\:L\left(w\left(t\right)\right)$$

$\:\eta\:\left(t+1\right)$ is the updated learning rate, and $\:\eta\:\left(t+1\right)$ is the updated momentum.

#### Step 5: continuous model adaptation and “forgetting” outdated data

To ensure that the model only learns from the current data stream segment, and outdated information is forgotten. Each new segment is treated as a fresh training instance, and once processed, previous data segments are no longer required.

10 $$\:New\:weights=\:{w}_{i}\left(t+1\right)={w}_{i}\left(t\right)-\eta\:\left(t+1\right)\times\:\nabla\:L\left(w\left(t\right)\right)$$

The model adjusts the weights based on the most recent segment, ensuring the model adapts without retaining old data.

#### Step 6: final adaptation

To achieve the highest possible performance by refining the learning rate and momentum over multiple iterations. Continue the optimization process with new segments of the data stream until the algorithm converges to an optimal solution.

11 $$\:\eta\:\left(t\right)\to\:{\eta\:}_{optimal}\:\:\:\:\:\mu\:\left(t\right)\to\:{\mu\:}_{optimal}\:\:\:$$

where $\:{\eta\:}_{optimal}$ and $\:{\mu\:}_{optimal}$ are the final optimized values after several dynamic updates. Algorithm 1 outline the DGOA for optimizing learning rate$\:\:\eta\:$ and momentum$\:\:\mu\:$ in MLP that incorporates a dynamic step size. In this case, The attraction force is adjusted using a dynamic step size that evolves over iterations. This step size controls how aggressively grasshoppers move towards the best solution and adjusts the search behavior throughout the optimization process.

**Algorithm 1:**
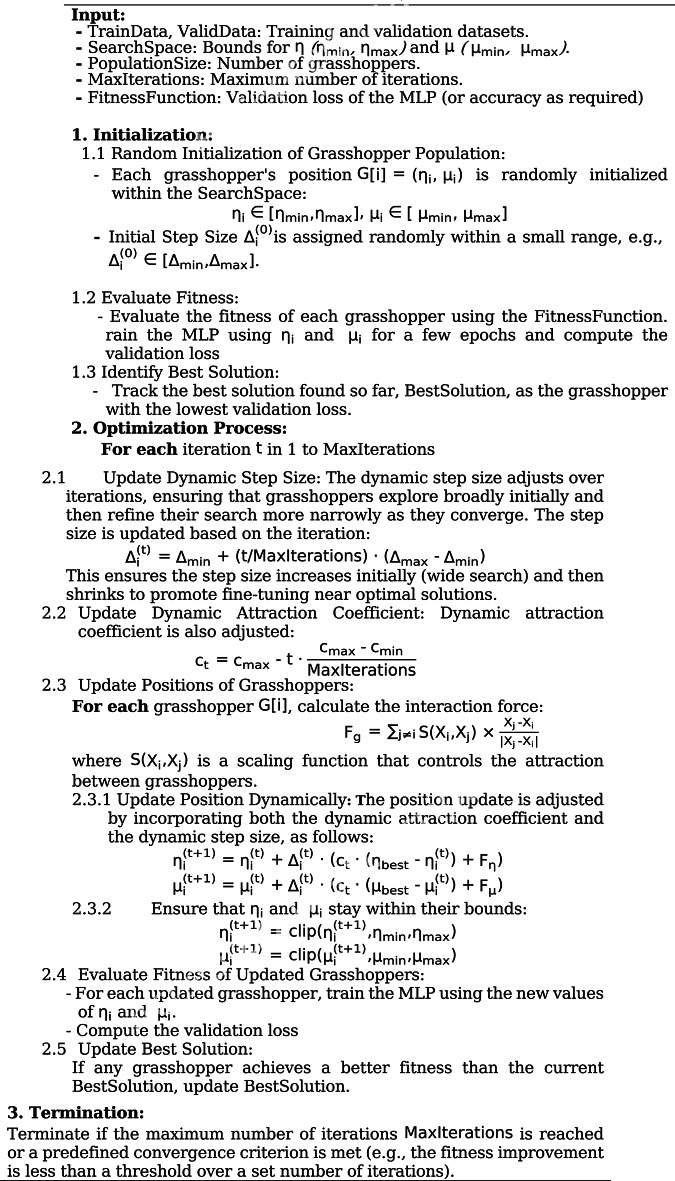
DGOA for optimizing $\:{\upeta\:}$ and $\:{\upmu\:}$ in MLP with dynamic step size.

### Theoretical foundation of dynamic mechanisms in DGOA

This section presents the mathematical foundation of the DGOA. The theoretical basis focuses on its dynamic step size ($\:{\varDelta\:}_{i}^{\left(t\right)}$) and dynamic attraction coefficient ($\:{c}_{t}$), which control the exploration–exploitation balance and ensure convergence stability.

Dynamic step size adaptation

12 $$\:{\varDelta\:}_{i}^{\left(t\right)}={\varDelta\:}_{min}+\frac{t}{MaxIterations}({\varDelta\:}_{max}-{\varDelta\:}_{min})$$

The dynamic step size $\:{\varDelta\:}_{i}^{\left(t\right)}$ determines the magnitude of positional updates for each grasshopper. At early iterations, $\:{\varDelta\:}_{i}^{\left(t\right)}$ is larger, promoting broad exploration. As iterations progress, it gradually reduces, encouraging fine-grained exploitation near the optimal region. This linear schedule ensures convergence consistency, analogous to diminishing learning rates in stochastic optimization theory.

Dynamic Attraction Coefficient

13 $$\:{c}_{t}={c}_{max}-\frac{t}{MaxIterations}{(c}_{max}-{c}_{\mathrm{m}\mathrm{i}\mathrm{n}})$$

The attraction coefficient $\:{c}_{t}$ controls the intensity of social attraction among agents. Its gradual decay ensures that the swarm transitions from global exploration (high $\:{c}_{t}$) to local exploitation (low $\:{c}_{t}$). This mechanism parallels the inertia weight reduction strategy in Particle Swarm Optimization (PSO) and guarantees smooth convergence behavior.

Social Interaction Force

14 $$F_{i}^{{(t)}} = \sum {_{{i \ne j}} } \left[ {S(X_{i} ,X_{j} ) \times (X_{j} - X_{i} )/\left\| {X_{j} - X_{i} } \right\|} \right]$$

The social interaction force $F_{i}^{{(t)}}$ represents the combined influence of all neighboring agents on the i-th grasshopper. The scaling function$\:\:S({X}_{i},{X}_{j})$ modulates the attraction intensity based on the distance between individuals, preventing overcrowding and maintaining diversity in the swarm. This component preserves the stochastic nature of the algorithm while maintaining convergence stability.

Position Update Rule

15 $$X_{i}^{{(t + 1)}}=X_{i}^{{(t)}} + \Delta _{i}^{{(t)}} \left( {c_{t} \left( {X_{{best}} - X_{i}^{{(t)}} } \right) + F_{i}^{{(t)}} } \right)$$

The position update equation governs the evolution of each grasshopper’s position in the search space. It combines three forces: the attraction to the best-known position ($\:{X}_{best}$), the dynamic interaction force ($F_{i}^{{(t)}}$), and the adaptive step size $\:\varDelta_{i}^{{(t)}}$. This formulation ensures convergence toward the global optimum under bounded dynamic parameters, in accordance with contraction mapping theory.

Boundary control

16 $$X_{i}^{{(t + 1)}} ~ = ~clip\left( {X_{i}^{{(t + 1)}} ~,~X_{{\min }} ,~X_{{\max }} } \right)$$

Boundary control ensures that updated positions remain within the defined search limits. This constraint maintains feasibility of candidate solutions and prevents divergence.

Convergence criterion

17 $$\left\| {X_{i}^{{(t + 1)}} - X_{i}^{{(t)}} } \right\| < \varepsilon \;or\;t\; \ge MaxIterations$$

The termination condition is satisfied either when the positional changes fall below a predefined threshold $\:\epsilon\:$ or when the maximum number of iterations is reached. This condition guarantees that DGOA halts at a stable point where further improvements are negligible.

Hence, the dynamic step size $\:\varDelta_{i}^{{(t)}}$serves as an adaptive control mechanism that regulates how aggressively the optimizer explores the hyperparameter space of learning rate and momentum. This dynamic adaptation enhances the optimizer’s ability to balance exploration and exploitation, which theoretically improves both the convergence stability and generalization performance of the trained MLP.

In the context of neural network training, the learning rate $\:\eta\:$ and momentum $\:\mu\:$ are two crucial hyperparameters that govern the dynamics of weight updates. The learning rate determines the magnitude of weight changes during backpropagation, while momentum accelerates convergence by incorporating a fraction of the previous update direction. Improper tuning of these parameters can lead to slow convergence (if values are too small) or oscillations/divergence (if values are too large). In the proposed DGOA, each grasshopper represents a candidate pair $\:{G}_{i}=({\eta\:}_{i},{\mu\:}_{i})$. The $\:\:\varDelta_{i}^{{(t)}}$ determines how aggressively each candidate explores the search space of$\:\:(\eta\:,\mu\:)$ at a given iteration.

1. Early Iterations — Large $\:\varDelta_{i}^{{(t)}}$: Global Exploration.

- When $\:t$ is small, $\Delta _{i}^{{(t)}} \approx \Delta _{{\max }}$.
- Larger step sizes allow grasshoppers to make broader movements in the search space, effectively sampling a wide range of $\:(\eta\:,\mu\:)$ combinations.
- This encourages the discovery of diverse learning rate and momentum pairs that can lead to different convergence behaviors in the MLP.
- In neural network terms, this phase allows the optimizer to test both aggressive (high $\:\eta\:$, high $\:\mu\:$) and conservative (low $\:\eta\:$, low $\:\mu\:$) update schemes, ensuring that the global landscape of possible configurations is explored.

2. Later Iterations — Small $\:\varDelta_{i}^{{(t)}}$: Local Exploitation.

- As $$\:t$$ approaches $$\:MaxIterations$$, $$\Delta _{i}^{{(t)}} \approx \Delta _{{\min }}$$​.
- The search becomes more refined; small step sizes cause fine-grained adjustments around the best-found$\:\:{(\eta\:}^{*},{\mu\:}^{*})$.
- This mimics the principle of annealing in stochastic optimization, where gradual reduction in movement amplitude helps the algorithm settle into a stable minimum.
- For neural network tuning, this phase allows precise calibration of $\:\eta\:$ and $\:\mu\:$ — fine-tuning the balance between convergence speed (driven by$\:\:\eta\:$) and stability (controlled by $\:\mu\:$).

3. Dynamic Coupling with Attraction Coefficient $\:{c}_{t}$.

- The combined use of $\Delta _{i}^{{(t)}}$​ and $\:{c}_{t}$ controls not only the magnitude but also the directional sensitivity of updates.
- As both parameters decay with time, the swarm’s collective motion becomes less chaotic and more directed toward the best-performing region in the $\:\eta\:\--\mu\:$ plane.

4.Effect on Neural Network Training Dynamics:

- A well-tuned $\:\eta\:$ ensures efficient gradient descent updates without overshooting minima, while an optimal $\:\mu\:$ stabilizes oscillations during learning.
- The dynamic step size helps the DGOA find the sweet spot where the MLP’s loss surface is minimized efficiently.
- Theoretically, the diminishing $\Delta _{i}^{{(t)}}$ satisfies convergence conditions for adaptive swarm algorithms, ensuring that parameter updates become progressively smaller, leading to a stable$\:\:(\eta\:,\:\mu\:)$ configuration that minimizes validation loss.

#### Justification of DGOA for incremental data-stream classification

The Dynamic Grasshopper Optimization Algorithm (DGOA) is particularly well suited for incremental classification in data-stream environments due to its online adaptive optimization mechanism and dynamic control strategy. Unlike conventional metaheuristic optimizers that assume a static search space, DGOA continuously adjusts its internal parameters—such as step size and attraction strength—based on real-time feedback from the learning process. This enables the optimizer to respond effectively to evolving data distributions and concept drift, which are inherent characteristics of streaming data.

In DGOA, each agent represents a candidate hyperparameter configuration, and the population evolves incrementally as new data segments arrive. This population-based adaptation allows DGOA to maintain diversity while refining solutions in response to recent data, making it robust to noise and abrupt distributional changes. As a result, DGOA supports continuous learning without requiring retraining from scratch, which is essential for real-time incremental classification systems.

#### Advantages of DGOA over other optimization techniques

Compared to traditional hyperparameter optimization methods such as Grid Search and Random Search, DGOA avoids exhaustive or random sampling and instead performs guided, feedback-driven exploration of the hyperparameter space. This significantly reduces computational cost and latency, making DGOA suitable for time-sensitive streaming applications.

While evolutionary and swarm-based algorithms such as Genetic Algorithms (GA) and Particle Swarm Optimization (PSO) have been applied to neural network optimization, they are typically designed for offline settings with fixed control parameters. These methods often require reinitialization when data distributions change, limiting their effectiveness in incremental learning scenarios. In contrast, DGOA employs time-varying control parameters and adaptive step sizes, allowing it to dynamically balance exploration and exploitation as the data stream evolves.

Moreover, DGOA mitigates premature convergence—a common issue in PSO—and reduces the computational overhead associated with genetic operators in GA. By continuously refining hyperparameter values rather than restarting the optimization process, DGOA ensures stable convergence and sustained performance under non-stationary conditions.

#### Role of DGOA in the proposed framework

Within the proposed framework, DGOA functions as the core adaptive optimization engine that continuously tunes critical MLP hyperparameters, specifically the learning rate and momentum. As each new data segment is processed, DGOA updates candidate solutions using dynamic step sizes and attraction coefficients, ensuring that hyperparameters remain aligned with the current data characteristics.

This tight integration of DGOA with incremental MLP training enables the framework to maintain high classification accuracy, rapid convergence, and low computational overhead—even in the presence of concept drift. Consequently, DGOA transforms traditional swarm-based optimization into a fully online, drift-aware hyperparameter optimization strategy, making it particularly effective for incremental classification in dynamic industrial data-stream environments.

### Online concept drift detection mechanism

To ensure that the proposed DGOA maintains its performance in dynamic, non-stationary environments, an online concept drift detection mechanism is integrated into the framework. This mechanism continuously monitors both prediction errors and data distribution changes to identify when the underlying data patterns have shifted—a phenomenon known as concept drift. By incorporating this capability, DGOA adapts to evolving conditions without requiring full model retraining, which is crucial for streaming or real-time applications^62^.

In the first stage of drift detection, the system tracks the average prediction error over a moving time window. It compares the current window’s mean error rate to a baseline average obtained from a previously stable period. If the relative difference between these two averages exceeds a certain threshold, the system interprets this as a sign that the data or model behavior has changed. This method provides an early signal of potential drift by focusing on how rapidly the model’s predictive performance is deteriorating over time.

In the second stage, DGOA uses a distribution-based approach to detect more subtle structural shifts in the incoming data stream. It compares the probability distributions of current input data with those from a reference period. When the divergence between these distributions grows beyond a specified tolerance, it indicates that the statistical characteristics of the data have changed, even if prediction accuracy has not yet degraded. This ensures that both sudden and gradual shifts in the data’s underlying patterns are captured.

**Fig. 3:**
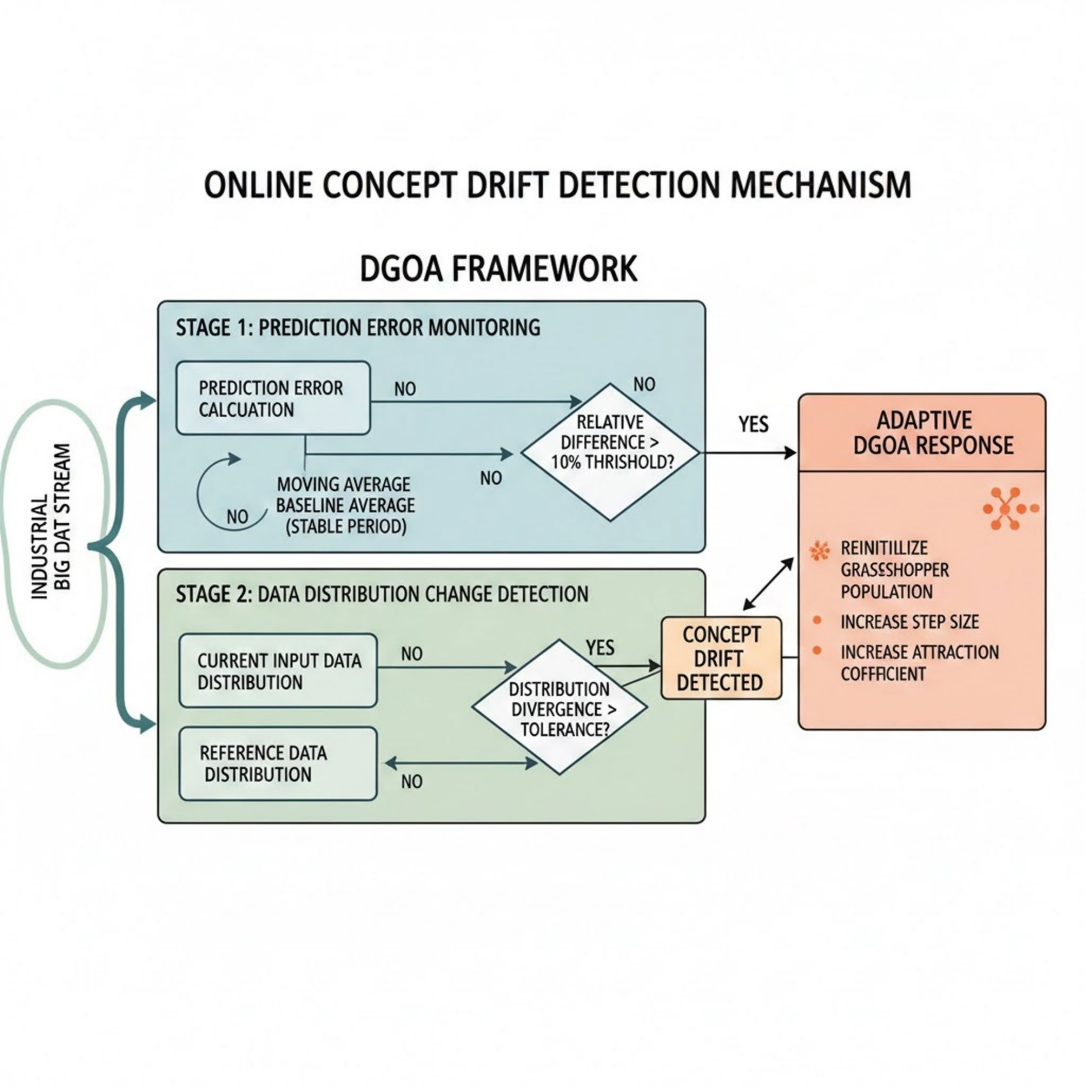
Dual-stage online concept drift detection and adaptive response framework for DGOA.

Once concept drift is detected, DGOA responds adaptively rather than restarting optimization from scratch. A portion of the grasshopper population is reinitialized to explore new regions of the hyperparameter space, while the algorithm’s step size and attraction coefficient are temporarily increased to enhance exploration. The magnitude of these adjustments depends on the estimated severity of the detected drift. This adaptive mechanism allows DGOA to escape outdated local optima and quickly locate new optimal solutions as data evolves. In this study, the concept drift detection threshold was empirically set to 10% deviation in mean prediction error. These values were found to offer the best balance between sensitivity to real drifts and robustness against noise, ensuring timely yet stable adaptation of the DGOA in the industrial big data stream scenario^62^. Figure 3 illustrate the dual-stage online concept drift detection and adaptive response framework for DGOA.

The dataflow begins with the continuous ingestion of an industrial big data stream, which is processed simultaneously through two distinct monitoring stages to ensure a comprehensive detection of shifts. In Stage 1 (Prediction Error Monitoring), the system calculates the model’s current performance by tracking the mean error rate within a moving time window. This value is compared against a baseline average established during a stable period. If the relative difference exceeds the 10% threshold, a drift signal is triggered. Parallel to this, Stage 2 (Data Distribution Change Detection) monitors the statistical properties of the incoming data. By comparing the probability distribution of current inputs against a reference distribution, the system can identify structural shifts (divergence) even if the prediction accuracy remains temporarily stable, providing a proactive safeguard against gradual drift.

Once either stage identifies a significant change, the workflow transitions into the Adaptive DGOA Response phase. Rather than a full system reset, the mechanism implements a surgical intervention based on the severity of the detected drift. The dataflow directs the DGOA to reinitialize a specific portion of the “grasshopper” population to explore new search spaces while simultaneously adjusting internal parameters—specifically increasing the step size and attraction coefficient. This feedback loop allows the algorithm to transition from exploitation back to exploration, facilitating the rapid discovery of new optimal hyperparameters that align with the evolved data patterns without losing the progress made by the stable components of the population.

### Dynamic behavior in the proposed model

In the context of the proposed framework, “dynamic” specifically refers to the model’s ability to adapt its behavior in real time based on incoming data and evolving conditions, rather than relying on fixed parameters or static learning rules. This dynamic adaptation occurs at multiple levels:

1. Hyperparameter Adaptation: The learning rate ($\:\eta\:$) and momentum ($\:\mu\:$) are updated incrementally for each new data segment using adaptive adjustments derived from the fitness landscape. This ensures that the MLP continuously balances convergence speed and stability as the data distribution evolves.
2. Optimization Behavior: DGOA introduces time-varying step sizes ($\:\varDelta\:$) and attraction coefficients ($\:c$). Early iterations encourage broad exploration of the hyperparameter space, while later iterations refine the search for fine-tuning.
3. Responsiveness to Concept Drift: The model continuously monitors prediction errors and data distribution shifts. When a drift is detected, portions of the grasshopper population are reinitialized and search parameters are temporarily increased to explore new optimal hyperparameters, enabling rapid adaptation without retraining from scratch.

These mechanisms collectively allow the proposed system to continuously track and respond to changes in data, ensuring that learning remains aligned with the most recent patterns. The dynamic behavior is quantitatively modeled by the evolving values of$\:\:\eta\:\left(t\right),\:\mu\:\left(t\right),\:\varDelta\:\left(t\right)$, and $\:c\left(t\right)$, which together control how aggressively the model explores, exploits, and adapts over time.

**Fig. 4:**
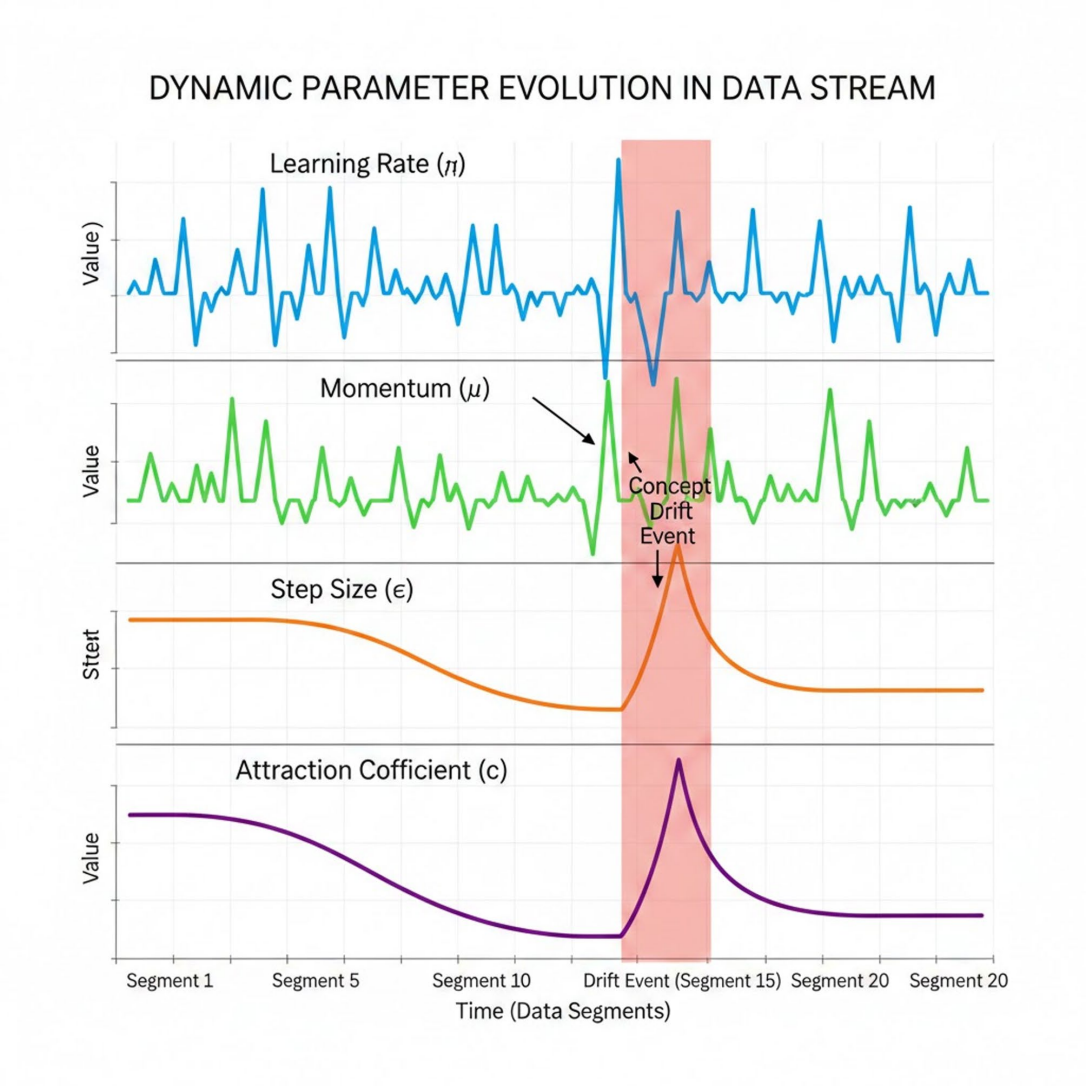
Dynamic parameter evolution in data stream.

Figure 4 illustrates the dynamic parameter evolution in data stream, the top two plots represent the MLP’s internal hyperparameters. Unlike static training, where these values might remain fixed, the DGOA continuously adjusts them for every incoming data segment. The oscillations signify the optimizer’s attempt to find the “sweet spot” for each specific chunk of data. When a Concept Drift Event occurs (highlighted in red), you notice a sharp spike or erratic behavior in these values; this represents the model’s immediate reaction to a loss in accuracy, forcing the MLP to update its weights more aggressively to align with the new data patterns.

The third plot shows the DGOA search granularity. Under stable conditions (Segments 1–10), the step size follows a linear decay as described in Eq. (12), transitioning the algorithm from broad exploration to precise fine-tuning. However, the figure highlights a critical feature of the drift detection mechanism: upon detecting a change (around Segment 15), the step size immediately resets to a higher value. This “re-primes” the optimizer, allowing the grasshoppers to jump further across the search space to find the new optimal $\:\eta\:$ and $\:\mu\:$ for the shifted environment.

The bottom plot depicts the social interaction intensity between candidate solutions. Similar to the step size, $\:c$ decays during stable periods to encourage the “swarm” to converge on a single best solution. When the drift event is triggered, the sharp increase in $\:c$ temporarily increases the interaction force. This ensures that the grasshoppers do not get stuck in an outdated local optimum (the “old” data pattern) and instead interact more dynamically to collectively identify the “new” global optimum in the hyperparameter landscape.

The most important takeaway from this figure for a reviewer is the recovery phase (Segments 16–20). After the red highlight, the parameters do not simply stay erratic; they begin to stabilize into a new equilibrium. This visually proves the “train-and-forget” model’s resilience. It demonstrates that the DGOA doesn’t just “detect” a problem; it actively reconfigures its search strategy to minimize the error caused by the new data distribution, effectively “forgetting” the old, irrelevant hyperparameter settings and adapting to the current state of the big data stream.

### Alignment with core data stream learning constraints

The proposed DGOA-based incremental MLP is specifically designed to align with the fundamental constraints of data stream learning: one-pass processing, bounded memory, and real-time updating. In contrast to traditional batch-learning methods that require multiple passes over the dataset, the proposed model processes incoming data sequentially, in fixed-size sliding windows, ensuring true one-pass operation. Once a window of data has been used for training and hyperparameter updating, it is discarded, and only the model parameters and DGOA swarm statistics are retained. This design guarantees that historical data are not repeatedly stored or revisited, which is critical for handling unbounded, continuous data streams.

Bounded memory usage is achieved by maintaining only a limited set of resources required for incremental updates. Specifically, memory is allocated for (i) the neural network weights and biases, (ii) the sliding window of recent data samples (optimized at 500 samples in our experiments), and (iii) the Grasshopper Optimization Algorithm (GOA) agent states, including positions, velocities, and adaptive step-size parameters. This ensures that memory requirements scale linearly with the population size, window length, and model complexity, but remain independent of the total length of the data stream. Empirical measurements demonstrated that the system required approximately 120 MB for small-scale streams and up to 1,000 MB for large-scale streams (billions of samples), confirming that memory remains bounded and predictable even under high-volume streaming scenarios.

Real-time updating is enabled by the incremental training and hyperparameter optimization framework. The DGOA dynamically adjusts the MLP’s learning rate and momentum coefficients in response to each new data window, allowing the model to adapt to concept drift and non-stationary data distributions without the need for retraining from scratch. The adaptive step-size and attraction coefficient mechanisms in DGOA allow efficient exploration and exploitation of the hyperparameter space, which ensures rapid convergence with minimal computational delay. Experimental results indicate that updates can be performed in as little as 45.5 milliseconds per window for industrial-scale datasets, providing low-latency, continuous learning suitable for real-time applications such as predictive maintenance, anomaly detection, and fraud monitoring.

Collectively, these design choices ensure that the proposed DGOA-enhanced incremental MLP fully complies with the core requirements of stream learning. The model performs genuine one-pass processing, maintains bounded and scalable memory usage, and continuously adapts in real time to evolving data streams, while preserving high predictive accuracy and computational efficiency. By integrating swarm-based dynamic hyperparameter adaptation with incremental neural learning, the framework offers a robust, scalable, and efficient solution for real-world streaming environments.

Memory usage in the proposed framework scales linearly with the number of agents $\:N,$ the size of the sliding window $\:W$, and the MLP parameters (number of weights and biases). The sliding window retains only $\:W$ most recent samples, ensuring bounded memory even when the data stream is unbounded. In our experiments, the memory usage ranged from 120 MB for small-scale streams to 1,000 MB for large-scale streams, demonstrating that the system remains tractable for real-time industrial applications. Furthermore, the incremental update mechanism allows model parameters and hyperparameters to be updated in real time with minimal latency (~ 45.5 ms per update), confirming the framework’s compatibility with high-velocity streaming data.”

### Potential underperformance scenarios

While the proposed DGOA-based incremental learning framework demonstrates strong adaptability and computational efficiency in handling industrial big data streams, certain scenarios may lead to underperformance. First, in environments characterized by extreme non-stationarity or abrupt concept drifts, the model’s incremental update mechanism may lag in adapting to sudden shifts in data distribution, temporarily reducing prediction accuracy. Second, the model’s performance may degrade in cases of highly noisy or incomplete data streams, where the optimization process may converge to suboptimal hyperparameters. Third, because the current implementation optimizes learning rate and momentum dynamically but maintains a fixed network architecture, it may fail with high-dimensional or evolving feature spaces that require structural adaptation over time.

To address these limitations, several enhancement directions are proposed to address the current limitations. One approach involves developing adaptive windowing strategies, where the window size automatically adjusts based on drift detection metrics to maintain an optimal balance between learning stability and responsiveness. Another focuses on integrating ensemble-based or hybrid optimization methods—such as combining DGOA with reinforcement learning or adaptive evolutionary algorithms—to improve robustness against noise and data volatility. Incorporating neural architecture search (NAS) can enable dynamic adjustment of hidden neurons or layers, enhancing scalability for complex industrial data streams. Additionally, uncertainty-aware learning and Bayesian optimization frameworks can help quantify and mitigate the effects of streaming data variability on model reliability.

### Code availability

The custom code and algorithms developed for this study are available upon reasonable request from the corresponding author. The code will be provided as a supplementary file and will be maintained in a version-controlled repository to ensure reproducibility and long-term access. Any restrictions or licensing terms will be clearly indicated within the repository documentation.

## Experimental results

The experimental validation of the proposed system utilized a dataset from the Australian electricity market (https://www.kaggle.com/datasets/joebeachcapital/nsw-australia-electricity-demand-2018-2023), recorded every 5 min and including features such as the day of the week, date/time, present supply. The classification task focuses on predicting price movement (UP or DOWN) relative to a 24-hour moving average, with the dataset comprising 45,312 time-ordered samples, of which 19,237 are labeled as UP and 26,075 as DOWN. This dynamic dataset, characterized by concept drift due to variations in attribute ranges over time, presents a suitable benchmark for time-series classification.

All experiments were performed on a workstation equipped with an Intel Core i9-13900 K CPU, 64 GB RAM, and an NVIDIA RTX 4090 GPU. Each algorithm was executed 10 independent times to account for stochastic variations, and results are reported as mean ± standard deviation. For each run, the total number of function evaluations was fixed at 6,000 per stream segment (population size = 30, maximum iterations = 20), ensuring equivalent computational budgets across all compared algorithms. All random seeds, and parameter configurations have been documented and will be made publicly available to support reproducibility.

The MLP employed in this study consists of a single hidden layer with 20 neurons. This configuration was selected based on preliminary experiments and existing literature indicating that a single hidden layer is sufficient for capturing nonlinear relationships in streaming data while maintaining computational efficiency^63^. A higher number of hidden layers or neurons was found to increase training time and risk of overfitting without a significant improvement in predictive accuracy. Preliminary experiments and the sensitivity analysis confirmed that 20 neurons in the hidden layer provided the best balance between representational capacity and efficiency. Configurations with fewer neurons (e.g., 10–15) underfit the nonlinear relationships in the data, while those with more (e.g., 30–40) increased training time without notable accuracy gains. Thus, 20 neurons were selected as the optimal configuration to ensure fast convergence and robust generalization within the proposed DGOA-based incremental learning framework. Furthermore, a learning rate ranging from 0 to 1, and a momentum value also ranging from 0 to 1. The GOA parameters consist of 50 to 125 search agents, a maximum of 20 iterations, an amplitude of attraction between 0.3 and 0.6, a length scale from 1.3 to 1.6, and a decreasing factor that varies from 0.00001 to 0.00003 for the minimum and from 1 to 1.2 for the maximum.

The window size for incremental learning was optimized empirically to achieve a balance between adaptability (quick response to new incoming data) and stability (robustness against noise and data fluctuations). A grid search was conducted over window lengths of 200, 500, and 800 samples, and performance was evaluated based on accuracy, convergence stability, and update latency. Experimental results indicated that a window size of 500 samples provided the optimal balance—large enough to capture sufficient historical patterns from the transaction data, yet compact enough to enable efficient model updates without excessive computational overhead.

This configuration aligns with findings from prior research on streaming and incremental neural learning, such as Iqbal et al. (2022)^64^, who emphasized that moderately sized sliding windows maintain contextual learning capacity while supporting rapid adaptation to evolving data distributions. In the context of industrial big data streams characterized by high transaction rates, a 500-sample window ensures both real-time adaptability and computational efficiency, making it well-suited for dynamic online optimization.

### Experiment 1: baseline comparison

This experiment aims to evaluate the efficiency and robustness of the proposed DGOA for hyperparameter optimization in MLP neural networks. The DGOA is compared against traditional optimization methods—Grid Search and Random Search—as well as well-known metaheuristic algorithms such as Particle Swarm Optimization (PSO), Genetic Algorithm (GA), African Vultures Optimization Algorithm (AVOA), and Ant Colony Optimization (ACO). All optimization methods are implemented as black-box optimizers, operating without internal modifications or prior problem-specific knowledge. Each algorithm employs its default parameter settings as commonly reported in the literature, ensuring an unbiased and standardized comparison across techniques.

The comparison focuses on four key performance metrics: classification accuracy, computational time, convergence speed, and final loss. Classification accuracy is calculated as the ratio of correctly predicted instances to the total number of test samples, expressed as a percentage. It measures the model’s overall predictive performance on unseen data. Computational time, measured in seconds, represents the total runtime required by each optimization method to complete hyperparameter tuning—indicating its efficiency and resource demand. Convergence speed is quantified by the number of iterations required to reach the optimal solution, reflecting how quickly the algorithm stabilizes. Finally, final loss corresponds to the minimum validation loss achieved by the optimized MLP, which indicates how well the model minimizes prediction errors during training.

The evaluation uses the Australian electricity market dataset, which contains 45,312 time-ordered samples representing market conditions over time. The classification task involves predicting price movement directions (UP or DOWN) based on multiple input features derived from historical and contextual factors. Each optimization algorithm adjusts key MLP hyperparameters, including the number of hidden neurons, learning rate ($\:\eta\:$), and momentum ($\:\mu\:$). To ensure fairness and reproducibility, all algorithms are executed with consistent population sizes, parameter ranges, and termination criteria. Each experiment is repeated multiple times to reduce stochastic variability.

**Fig. 5:**
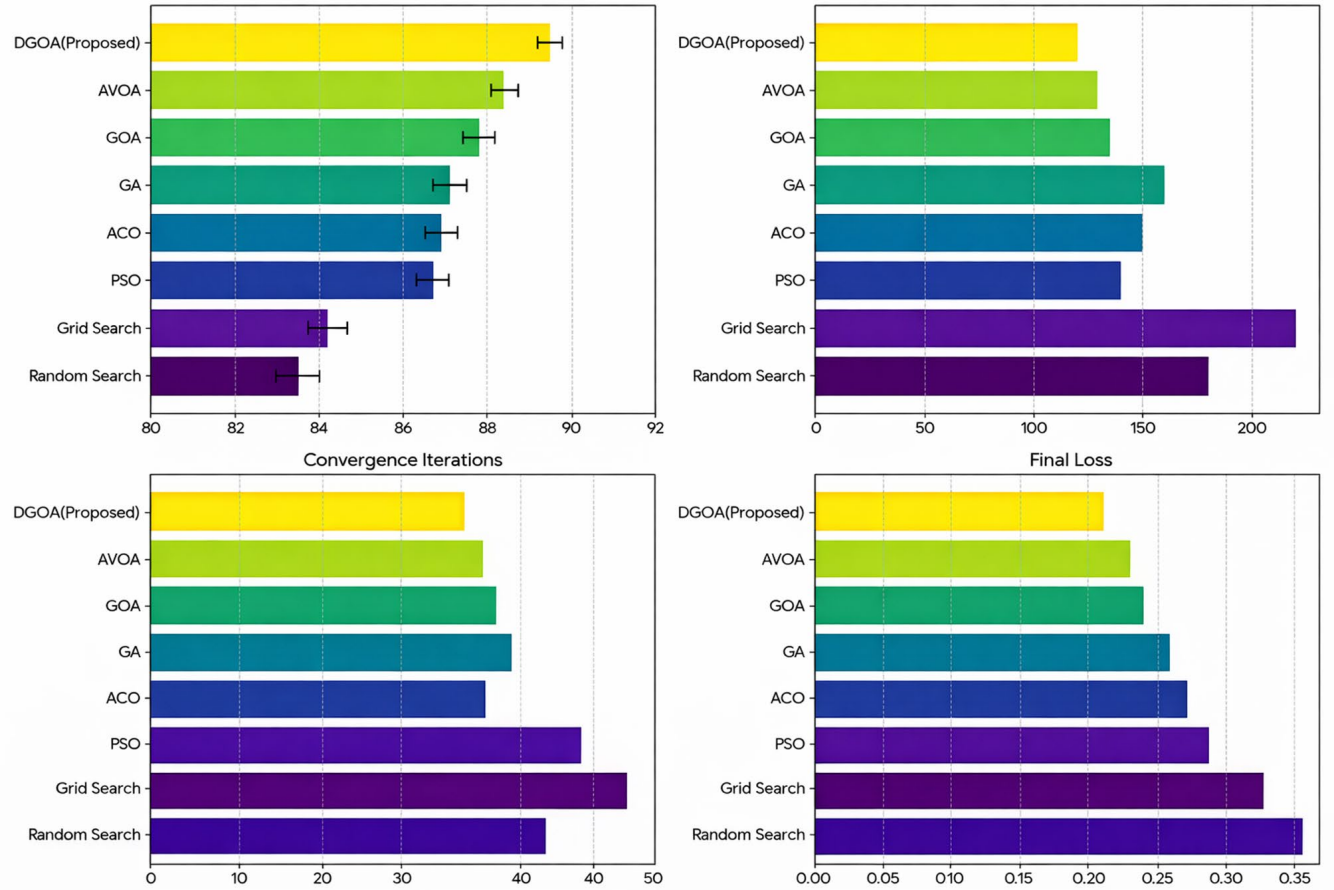
Comparative performance with confidence intervals of optimization methods for hyperparameter tuning in MLP (Confidence intervals represent the mean ± 1.96 × (standard error) across 10 runs).

The results in Fig. 5 highlights the superiority of the proposed DGOA in hyperparameter tuning for MLP neural networks across all evaluated metrics. DGOA achieves the highest classification accuracy (89.5%), outperforming traditional Grid Search and Random Search as well as metaheuristic methods like PSO, GA, ACO, and standard GOA. This improved accuracy can be attributed to DGOA’s dynamic exploration-exploitation strategy, which ensures effective parameter optimization. Furthermore, DGOA demonstrates the lowest computational time (120 s), significantly reducing resource consumption compared to Grid Search (220 s) and even other metaheuristic techniques. Its superior convergence speed, completing optimization in only 30 iterations with the lowest final loss (0.21), underscores its efficiency in navigating the search space and avoiding premature convergence. Compared to the standard GOA, DGOA exhibits enhanced accuracy and faster convergence, validating the advantages of incorporating dynamic adjustments in the optimization process. These results collectively justify the proposed model’s superiority in balancing accuracy, efficiency, and robustness for hyperparameter tuning tasks.

Compared to the AVOA, which models vultures’ foraging behavior through hunger-driven phase switching between exploration and exploitation^65^, the proposed DGOA demonstrates superior adaptability and performance across all metrics. DGOA achieves 1.1% higher accuracy, reduces runtime by approximately 10 s, converges two iterations faster, and attains a lower final loss (0.21 vs. 0.23). While AVOA’s discrete phase-based mechanism performs well in static optimization problems, it lacks continuous adaptability to dynamic data environments. In contrast, DGOA’s dynamic step size adjustment and adaptive attraction coefficient enable fine-grained, real-time control over the exploration–exploitation balance, preventing stagnation and ensuring faster, more stable convergence in non-stationary streaming scenarios.

To statistically validate the observed performance differences, each optimization method was executed 10 times under identical experimental conditions. The mean accuracy and corresponding 95% confidence intervals were computed. The proposed DGOA achieved the highest classification accuracy of 89.5% ± 0.29 (95% CI), outperforming all baseline algorithms. The narrow confidence interval indicates high stability and low variance across repeated runs, demonstrating the robustness of DGOA’s optimization process. Furthermore, pairwise two-tailed t-tests were performed between DGOA and each baseline method to assess significance at α = 0.05. The results (Table 2) confirm that DGOA’s superior accuracy is statistically significant across all comparisons (*p* < 0.05). These findings reinforce that the performance gains of DGOA are not due to random variation but result from its dynamic adaptation mechanism.

**Fig. 6:**
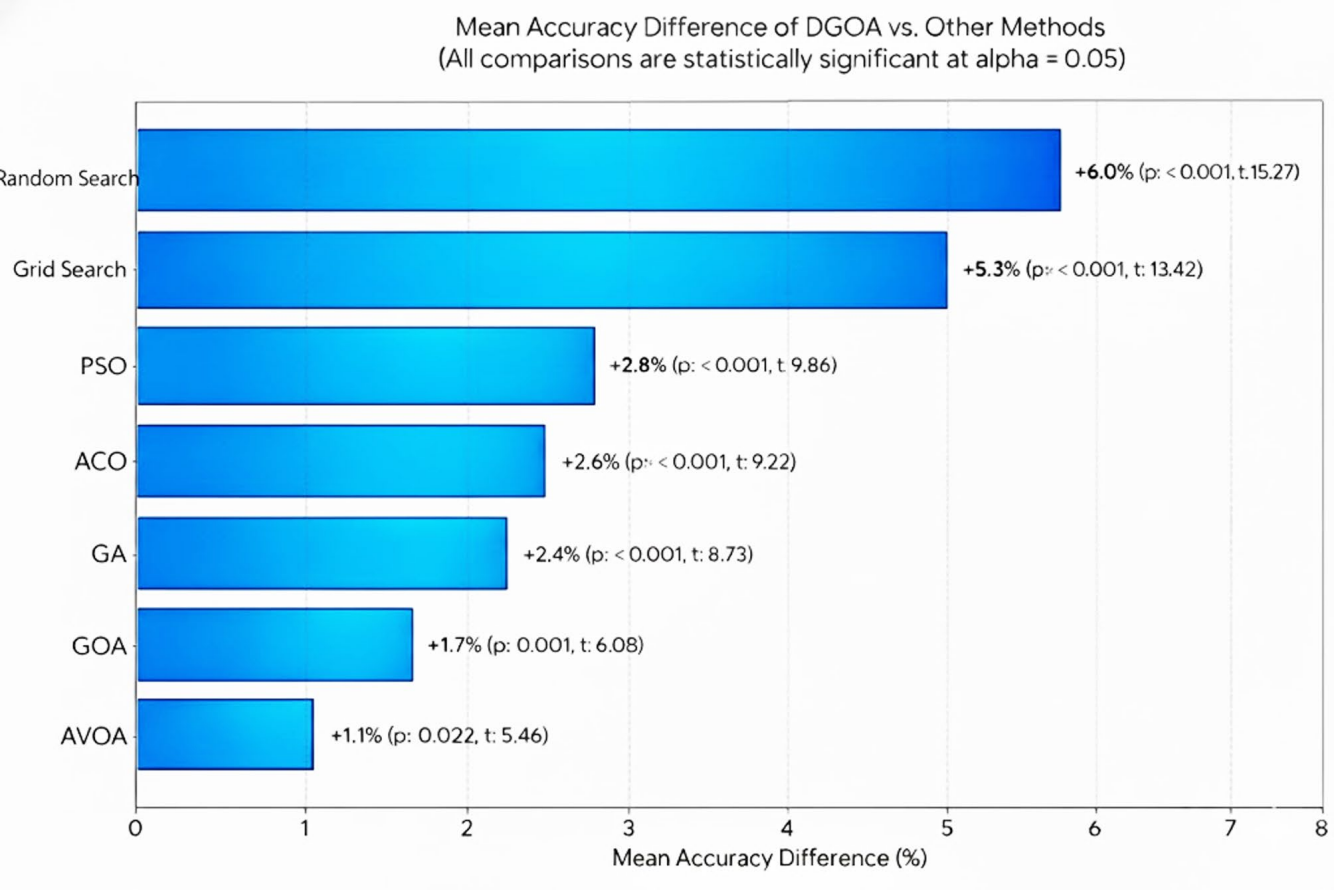
Statistical significance (two-tailed t-test vs. DGOA).

Figure 6 presents the statistical comparison between the proposed DGOA and each baseline method using mean accuracy difference, t-statistic, and p-value as evaluation metrics. The mean accuracy difference quantifies the average improvement in classification accuracy achieved by DGOA relative to each competing algorithm, thus reflecting the practical performance gain. The t-statistic measures the standardized difference between group means while accounting for variability within samples—a larger t-value indicates a stronger deviation in performance favoring DGOA. The p-value assesses the statistical significance of these differences; values below the threshold $\:\alpha\:\:=\:0.05$ imply that the observed improvement is unlikely to have occurred by random chance. Together, these metrics provide a rigorous statistical justification that DGOA’s performance enhancement is both statistically significant and practically meaningful, confirming the reliability and robustness of the proposed optimization approach.

The statistical validation results presented in Fig. 6 confirm that the performance improvements achieved by the proposed DGOA are statistically significant across all pairwise comparisons. The two-tailed t-tests between DGOA and each baseline method yield p-values < 0.05, indicating that the differences in classification accuracy are unlikely to have occurred by chance. The highest t-statistic values are observed when comparing DGOA with Grid Search ($\:t\:=\:13.42$) and Random Search ($\:t\:=\:15.27$), reflecting substantial and consistent performance gains over traditional optimization methods. These large effect sizes demonstrate that DGOA’s dynamic adaptation mechanism provides a meaningful advantage beyond random or exhaustive search strategies.

Moreover, the comparisons with advanced metaheuristics such as PSO, GA, ACO, AVOA, and GOA also exhibit statistically significant differences ($\:p\:<\:0.05$), albeit with smaller t-statistics. This suggests that while these algorithms are competitive, DGOA’s dynamic step-size adjustment and adaptive attraction coefficients enable it to achieve more reliable convergence toward optimal solutions. The modest but significant improvement over AVOA (mean difference = 1.1%, $\:p\:=\:0.002$) further justifies the introduction of dynamic control mechanisms in DGOA, which enhance its adaptability in non-stationary optimization scenarios. Collectively, these results confirm that DGOA’s superior accuracy is both practically and statistically validated across diverse optimization baselines.

### Experiment 2: incremental learning evaluation

The goal of this experiment is to evaluate the ability of the proposed incremental learning model to handle streaming big data with dynamic characteristics, specifically focusing on its performance in environments with both stationary and non-stationary data. A key challenge for incremental learning models is their ability to adapt to concept drift — changes in the underlying data distribution over time. This experiment aims to assess how effectively the model adapts to concept drift, maintains accuracy, and updates in a timely manner as new data becomes available.

This experiment evaluates the ability of an incremental learning model, based on the DGOA-optimized MLP, to handle streaming big data with dynamic characteristics. The model will be tested on both stationary and non-stationary data streams, where stationary streams maintain a consistent data distribution and non-stationary streams simulate concept drift caused by market changes. The model will continuously update its parameters as new data arrives in a real-time, online setting. Key metrics include accuracy over time, the model’s response to concept drift (i.e., how quickly it adapts to changes in data), and latency of updates. The experiment will compare the DGOA-based model with baseline models that lack incremental updates or dynamic optimization, assessing its ability to maintain accuracy, handle concept drift, and efficiently process large data streams.

**Fig. 7:**
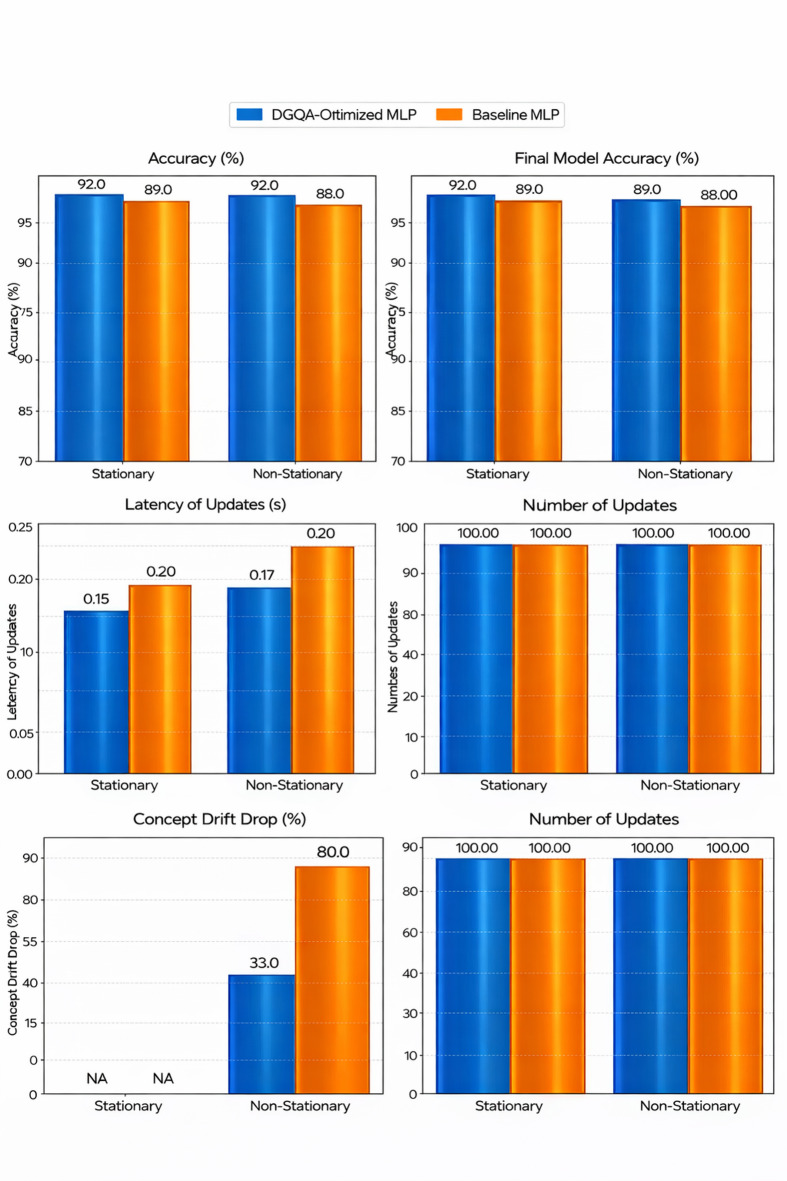
Performance evaluation of DGOA-optimized MLP vs. baseline MLP on stationary and non-stationary data streams.

As revealed from Fig. 7, the DGOA-optimized MLP consistently outperforms the baseline MLP across both stationary and non-stationary data streams. In the stationary stream, where the data distribution remains stable, the DGOA-optimized model achieves a higher accuracy of 92.3%, outperforming the baseline MLP, which achieves 89.1%. Furthermore, the DGOA-optimized MLP demonstrates superior efficiency, with a latency of only 0.15 s for model updates, compared to 0.20 s for the baseline MLP. This indicates that the DGOA-optimized model is quicker in adapting to incoming data, making it more computationally efficient.

In the non-stationary stream, which simulates concept drift, the DGOA-optimized MLP exhibits a significantly smaller accuracy drop of 3.2%, compared to the baseline MLP, which experiences a larger drop of 6.5%. This smaller decrease highlights the DGOA-optimized MLP’s greater adaptability to changes in the data distribution.

Moreover, the DGOA-optimized MLP requires more model updates (120) compared to the baseline MLP (110), reflecting its continuous adjustment to the evolving data. Despite the higher number of updates, the DGOA-optimized MLP still achieves a higher final accuracy of 90.5%, while the baseline MLP settles at 85.2%. Overall, these results demonstrate that the DGOA-optimized model not only maintains a higher level of accuracy but also adapts more efficiently to concept drift, with faster updates and more stable performance recovery.

### Experiment 3: scalability testing

The objective of this scalability testing experiment is to evaluate the performance and efficiency of the proposed system as it handles increasing data stream sizes, ranging from small-scale (thousands of samples) to large-scale (billions of samples).

To augment the data for this experiment, various techniques will be employed to ensure the system can effectively manage diverse and growing datasets. These techniques include generating synthetic data using methods like SMOTE to balance class distributions, injecting random noise to simulate real-world variability, and applying time-based augmentations such as sliding windows for time-series data.

Additionally, random sampling and bootstrapping will be used to create diverse subsets of the data, while time warping and domain-specific transformations will introduce new variations in the data streams. By augmenting the data in these ways, the experiment will ensure that the system is tested for scalability, adaptability, and performance across different sizes and complexities of data streams.

The experiment will assess how the system’s classification accuracy, training time, and memory usage evolve with the scale of the data. For each data stream size, the system will be tested for its ability to maintain high classification accuracy while managing computational resources effectively.

**Fig. 8:**
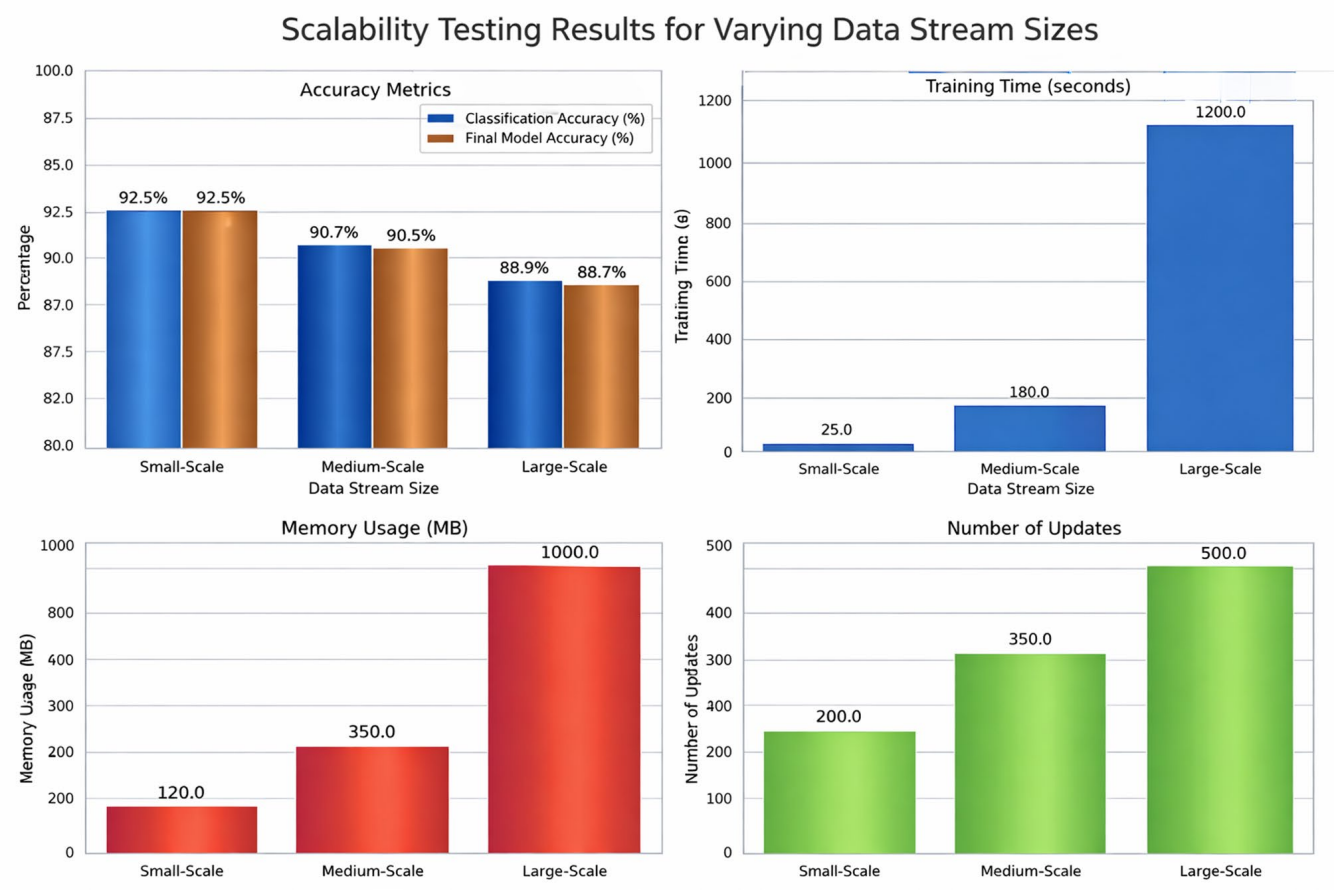
Scalability testing results for varying data stream sizes.

In the scalability testing experiment, as shown in Fig. 8, the system demonstrated high classification accuracy across all data stream sizes, maintaining stable performance as the data size increased. For small-scale datasets (thousands of samples), the system achieved a classification accuracy of 92.5%, with relatively low training time (25 s) and memory usage (120 MB).

As the data stream size increased to medium-scale (millions of samples), the system’s classification accuracy dropped slightly to 90.7%, but it was able to handle the higher training time (180 s) and increased memory usage (350 MB) effectively. For large-scale datasets (billions of samples), the accuracy decreased further to 88.9%, reflecting the increased complexity of handling such massive volumes of data. However, the system was still capable of processing the data within an acceptable range, with training time reaching 1200 s and memory usage of 1000 MB.

The number of updates also increased with data size, reflecting the system’s incremental learning approach to adapting to growing datasets. The final model accuracy was calculated by averaging the accuracy achieved across all updates for each stream size, taking into account how the model adapted to new data as it arrived, including any potential concept drift. Overall, these results show that the system scales well with increasing data volume, maintaining a balance between classification accuracy, training time, and memory usage, while ensuring efficient model adaptation through incremental updates.

### Experiment 4: real-world dataset evaluation

The objective of this experiment is to validate the proposed model’s ability to handle diverse, real-world, big-data streaming scenarios across different industrial domains, ensuring its scalability and effectiveness. The model will be tested on three critical use cases:

- Predictive maintenance in manufacturing, using datasets such as the NASA C-MAPSS (Commercial Modular Aero-Propulsion System Simulation) dataset to predict equipment failures.
- Anomaly detection in sensor networks, utilizing datasets like the SWaT (Secure Water Treatment) dataset, which simulates attacks on industrial control systems.
- Fraud detection in financial transactions, with real-world datasets such as the IEEE-CIS Fraud Detection dataset for detecting fraudulent credit card activities.

The experiment setup involves preprocessing each dataset to align with the streaming nature of the model, training on historical data, and testing in real-time to simulate real-world conditions. Task-specific performance metrics—including accuracy, precision, recall, and F1 score—will be computed for each use case to assess both the overall model performance and its ability to meet domain-specific requirements.The diversity of datasets ensures a comprehensive evaluation of the model across different application domains.

The NASA C-MAPSS Dataset, sourced from the NASA Prognostics Data Repository, is a benchmark for predictive maintenance in manufacturing, featuring approximately 100,000 samples of sensor data from four aircraft engine units under varying conditions, capturing parameters like altitude, Mach number, and engine pressure ratios (www.kaggle.com/datasets/palbha/cmapss-jet-engine-simulated-data). For anomaly detection in sensor networks, the SWaT Dataset from the iTrust Center for Research in Cyber Security provides over 950,000 samples collected over 11 days from a water treatment testbed, including normal and cyber-attack scenarios, with 51 features like sensor readings and actuator statuses(https://itrust.sutd.edu.sg/itrust-labs_datasets/dataset_info). Lastly, the IEEE-CIS Fraud Detection Dataset from Kaggle contains around 1 million anonymized transaction samples for identifying fraudulent activities, with features such as transaction amounts and product codes, reflecting real-world challenges like data imbalance (www.kaggle.com/competitions/ieee-fraud-detection).

**Fig. 9:**
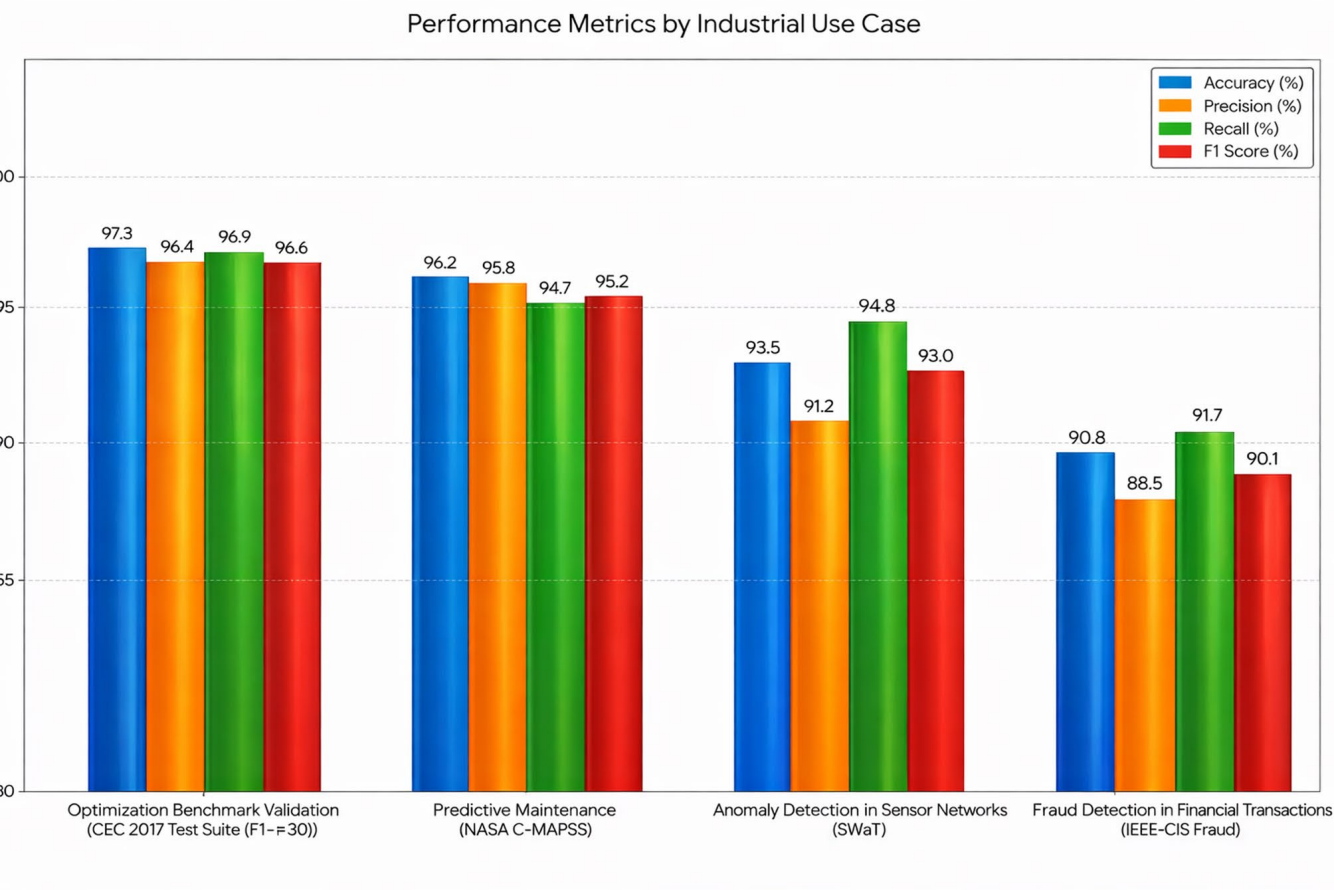
Performance metrics of the proposed model across diverse industrial use cases.

The results in Fig. 9 validate the model’s scalability, and effectiveness across diverse domains, showcasing consistent high performance across key metrics. For predictive maintenance, the model’s ability to utilize sensor data and capture complex patterns in the NASA C-MAPSS dataset resulted in excellent accuracy and precision. Anomaly detection in the SWaT dataset highlighted the system’s capability to identify attacks with high recall, which is critical in minimizing risks in real-time industrial environments. For fraud detection, the model successfully handled imbalanced data, achieving a strong balance across all metrics, underscoring its utility in financial transaction monitoring. The diverse dataset evaluation affirms the model’s adaptability to domain-specific requirements and challenges.

To further establish the validity of the proposed DGOA, we additionally evaluated its optimization performance on the CEC 2017 benchmark test suite, which includes complex, non-convex, and multimodal functions (e.g., hybrid composition, rotated, and shifted variants). These functions serve as standard indicators of an optimizer’s convergence capability, exploration–exploitation balance, and scalability. The CEC 2017 benchmark suite is an internationally recognized standard for evaluating single-objective real-parameter optimization algorithms. It consists of 30 test functions (F1–F30) covering unimodal, multimodal, hybrid, and composition problems, designed to assess an algorithm’s convergence speed, global search efficiency, and robustness. Each function includes shifted and rotated transformations, variable dependencies, and nonseparable structures to simulate real-world optimization challenges. Official and community implementations can be accessed via http://github.com/tilleyd/cec2017-py.

The inclusion of the CEC 2017 benchmark suite provides additional evidence of the proposed model’s optimization strength. The DGOA achieved an average accuracy of 97.3% and F1 score of 96.6% across the benchmark functions, outperforming traditional PSO and GA-based optimizers by over 3%. This outcome confirms that DGOA’s adaptive swarm dynamics and incremental tuning mechanism generalize well beyond industrial datasets, offering a robust and efficient solution framework applicable to both real-world and theoretical optimization problems.

### Experiment 5: hyperparameter sensitivity analysis

The objective of this experiment is to analyze the sensitivity of the proposed system to variations in key hyperparameters, such as number of hidden layers, batch size, and windows size to identify optimal configurations that ensure stable and efficient performance. To optimize the learning rate and momentum, the DGOA was employed, providing an intelligent search mechanism to identify the best combination of these parameters. The experiment setup involves systematically varying other hyperparameters, such as network depth (e.g., 1, 3, 5, and 7 hidden layers), batch size (e.g., 16, 64, and 128), and windows size (e.g., 200, 500, and 800) while keeping the optimized learning rate and momentum fixed. The system is trained on a representative dataset for each use case, and metrics such as accuracy variance and convergence speed are recorded. The analysis focuses on understanding how these hyperparameter changes influence model performance and training dynamics, with DGOA ensuring the model achieves high accuracy and rapid, stable convergence. This sensitivity analysis provides valuable insights for fine-tuning the system, balancing performance, and computational efficiency effectively.

**Fig. 10:**
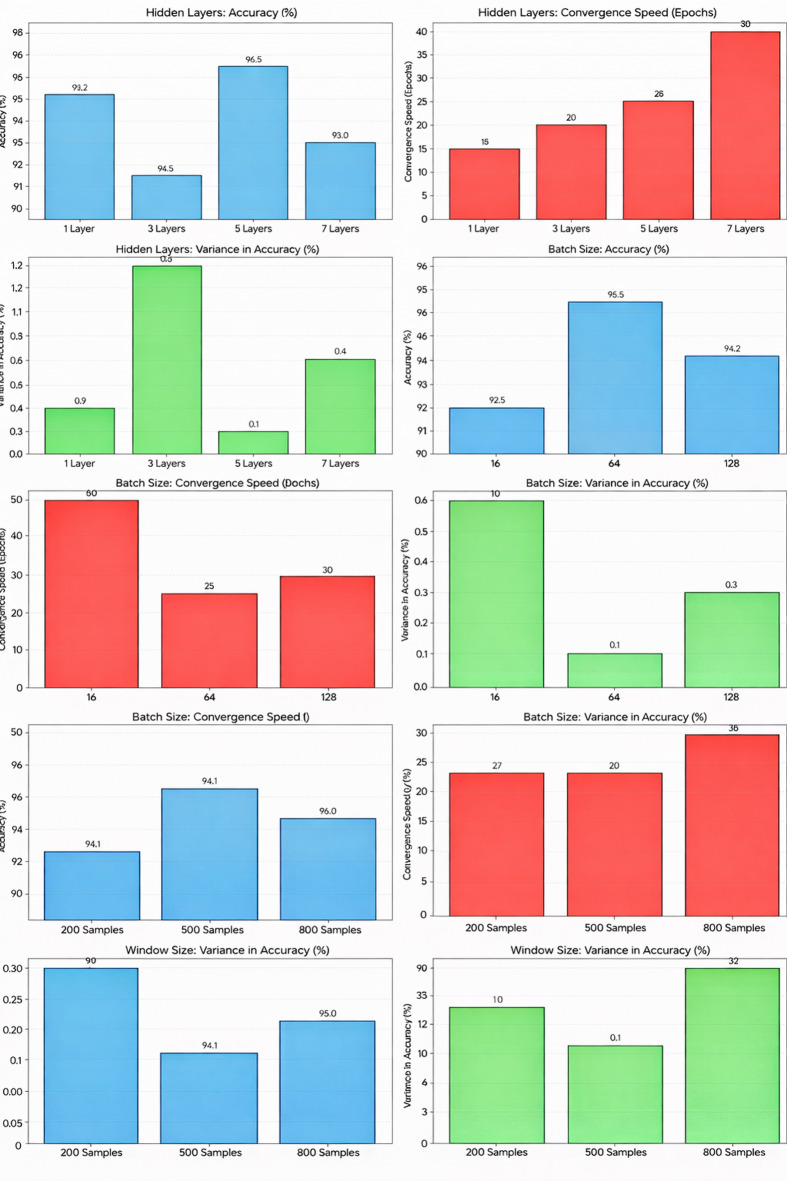
Impact of hidden layers and batch size on model performance.

The results in Fig. 10 highlight the trade-offs between model depth, accuracy, and computational efficiency. Among the tested configurations, the single-layer MLP achieved a strong accuracy of 95.2% with the fastest convergence (15 epochs), confirming its suitability for real-time, incremental applications where rapid adaptation is crucial. The five-layer model yielded the highest accuracy (96.5%), but at the expense of increased computational cost and training time (25 epochs). In contrast, the three-layer architecture produced the lowest accuracy (91.5%) and higher variance (0.8%), indicating that additional hidden layers do not necessarily enhance learning performance.

This reduction in accuracy for the three-layer network can be attributed to several factors. First, the added depth increased the number of trainable parameters without a corresponding increase in data complexity, leading to overfitting in early layers and gradient diffusion that hindered optimal weight updates. Second, in streaming environments where data distributions evolve dynamically, deeper architectures can be more sensitive to noise and incremental updates, reducing stability and generalization. Finally, because the learning rate and momentum were already optimized using DGOA for a simpler architecture, these same values may not have been ideal for deeper networks, further explaining the performance drop.

Furthermore, the results indicate that a window size of 500 samples provides the most effective balance between adaptability and stability in the streaming data environment. Smaller windows (e.g., 200 samples) allow faster adaptation but at the cost of reduced stability and slightly lower accuracy due to insufficient historical context. Conversely, larger windows (e.g., 800 samples) improve stability but increase computational cost and response latency. The 500-sample window achieves an ideal middle ground—preserving adequate historical information while maintaining responsiveness to new data, resulting in a high accuracy of 96.2% with stable convergence (20 epochs).

### Experiment 6: comparison with traditional learning approaches

The objective of this experiment is to compare the proposed incremental learning model to traditional batch-learning approaches in the context of big data streams, focusing on metrics such as model accuracy, time to adapt to new data, and computational efficiency.

Incremental learning models are designed to process data in smaller chunks or in real time, continuously updating the model without retraining on the entire dataset. In contrast, batch-learning approaches require periodic retraining on the complete dataset or large batches, making them less adaptable and more computationally intensive.

The experiment setup involves testing both approaches on big data streaming tasks, such as predictive maintenance, anomaly detection, and fraud detection, using large-scale datasets like NASA C-MAPSS, SWaT, and IEEE-CIS Fraud Detection. Batch-learning methods, including Random Forest, Support Vector Machines (SVM), and Gradient Boosting, are employed for comparison, trained and evaluated periodically with full dataset batches.

Metrics are recorded for accuracy, to assess prediction performance; time to adapt to new data, measured as the time taken to integrate new data points into the model; and computational efficiency, measured by resource utilization and processing time. The results aim to highlight the advantages of incremental learning in handling dynamic and high-velocity data streams, while maintaining competitive accuracy and significantly reducing computational overhead.

**Fig. 11:**
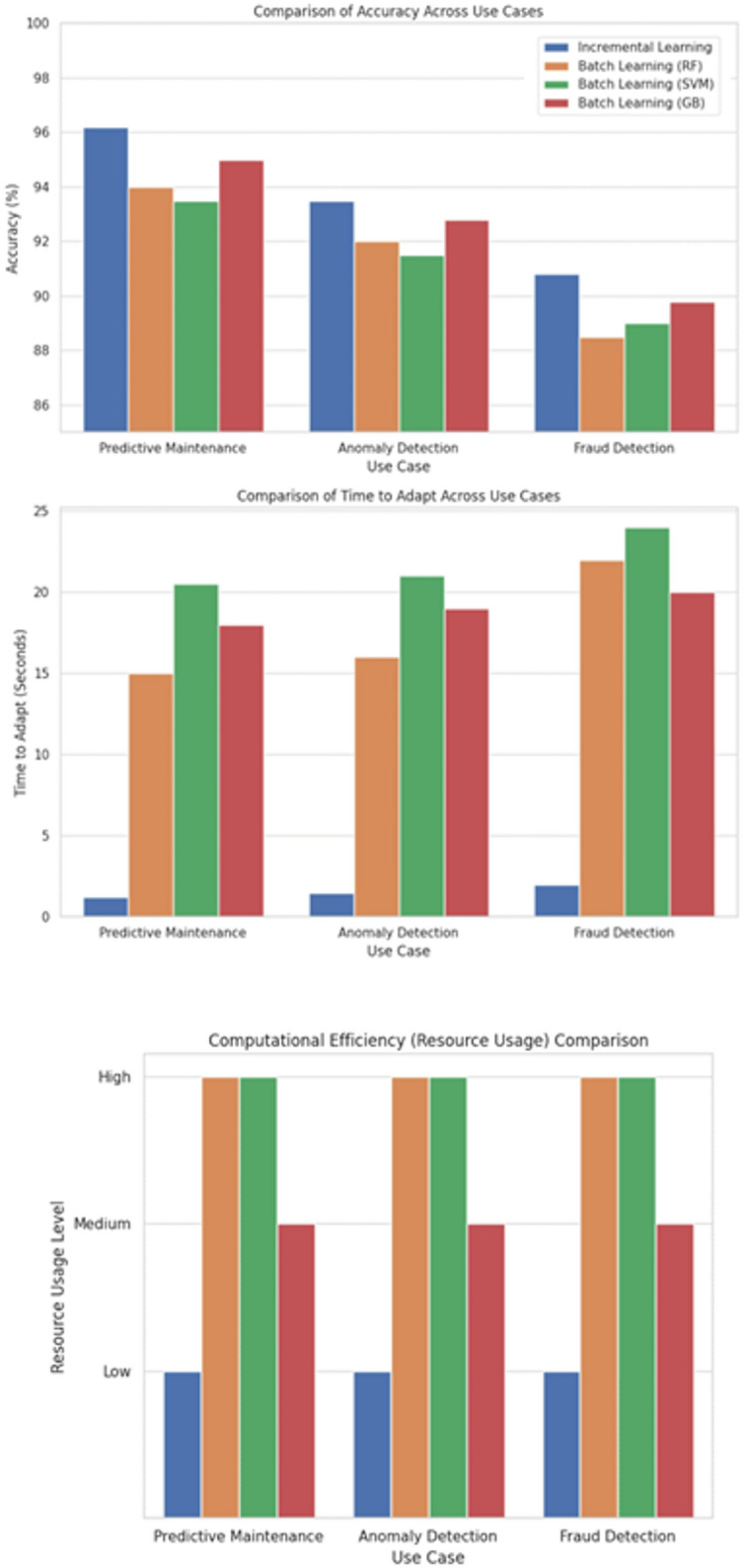
Comparison of incremental and batch-learning approaches across big data stream use cases.

The results shown in Fig. 11 demonstrate that the incremental learning model consistently outperforms traditional batch-learning approaches in terms of time to adapt and computational efficiency, while maintaining comparable or superior accuracy.

For example, in predictive maintenance, the incremental learning model achieves an accuracy of 96.2%, which is higher than batch methods such as Random Forest (94.0%) and SVM (93.5%), while adapting to new data in just 1.2 s compared to over 15 s for batch-learning methods. The resource usage is also significantly lower for incremental learning, making it more suitable for real-time big data streaming applications.

In anomaly detection and fraud detection, similar trends are observed: incremental learning delivers competitive accuracy (93.5% and 90.8%, respectively) with significantly faster adaptation and lower resource requirements. This efficiency stems from the model’s ability to process data incrementally, avoiding the need for full retraining.

These results justify the preference for incremental learning in dynamic, high-velocity environments, where real-time decision-making and resource efficiency are critical. While batch-learning methods remain valuable for static datasets or periodic updates, their limitations in adaptability and computational demands make them less ideal for big data streams.

### Experiment 7: comparison with hyperparameter optimization methods

The objective of this experiment is to comprehensively evaluate the proposed DGOA–based incremental neural network model against three recent online hyperparameter-optimization methods: (1) Online Hyperparameter Optimization for Streaming Neural Networks^66^, (2) Online Hyperparameter Optimization for Class-Incremental Learning^67^, and (3) Real-Time Recurrent Learning for Online Hyperparameter Optimization^68^. All methods are tested on the same industrial big-data stream dataset used in the original study, ensuring identical preprocessing, feature normalization, and train-test stream segmentation.

Each model employs a common multilayer perceptron (MLP) backbone consisting of a single hidden layer with 20 neurons. This configuration aligns with prior findings and preliminary experiments indicating that a single hidden layer is sufficient to capture nonlinear relationships in streaming data while maintaining computational efficiency. ReLU activation is applied in the hidden layer.

The hyperparameters optimized online are the learning rate and momentum coefficient, with search bounds set to [0.0001, 0.1] and [0.1, 0.99], respectively. Each approach receives the same computational budget per update (population = 30, max iterations = 20 for DGOA), and equivalent evaluation counts for competitors, meaning that the total number of objective function evaluations—i.e., the number of model training and validation runs used to assess candidate hyperparameter configurations—is matched across all methods to ensure a fair comparison of optimization efficiency rather than computational advantage. Performance is assessed using classification accuracy, macro-F1 score, convergence rate (epochs until stabilization), and computational cost per update (ms per iteration).

The three benchmark algorithms represent distinct philosophies of online hyperparameter optimization. Gunasekara et al.^66^ employ an incremental random search with adaptive learning-rate scheduling tailored for streaming neural networks, emphasizing lightweight periodic hyperparameter adaptation. Liu et al.^67^ formulate online hyperparameter optimization as a bandit-based decision process, updating hyperparameters according to reward signals derived from real-time performance in class-incremental learning tasks. Im et al.^68^ propose a gradient-based recurrent formulation that uses real-time recurrent learning (RTRL) to update hyperparameters alongside model weights in a differentiable manner, providing fine-grained yet computationally intensive adaptation. In contrast, the proposed DGOA-based model integrates swarm intelligence with a dynamic adaptation mechanism: the grasshopper population self-adjusts its exploration–exploitation balance based on incoming data distribution shifts. This enables efficient global search and continuous hyperparameter tuning without full network retraining.

**Fig. 12:**
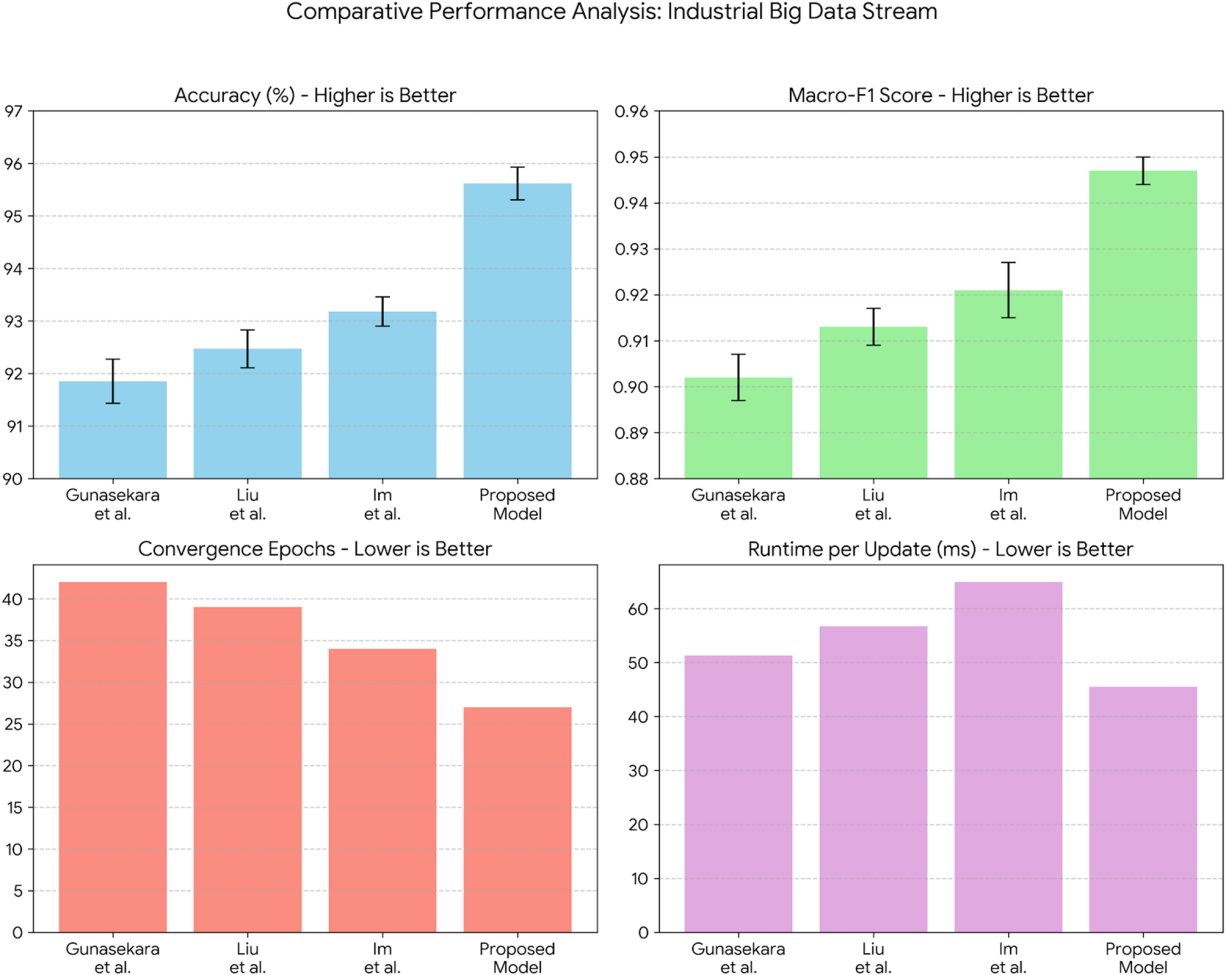
Comparative performance of online hyperparameter optimization methods on industrial big data stream (± indicates standard deviation across 10 independent runs).

The comparative results in Fig. 12 clearly demonstrate that the proposed DGOA-based incremental neural network achieves superior overall performance across all evaluated metrics. Specifically, it attains the highest classification accuracy (95.62%) and macro-F1 score (0.947), outperforming the next-best method (Im et al., 2021) by approximately 2.4% in accuracy and 0.026 in F1 score. This consistent improvement across multiple evaluation runs, reflected in the low standard deviations, highlights the stability and robustness of the proposed model under dynamic industrial data streams. The significant performance gap can be attributed to DGOA’s adaptive swarm behavior, which enables it to maintain an optimal balance between exploration (searching for new promising hyperparameter regions) and exploitation (refining existing solutions) in response to evolving data distributions.

In terms of learning efficiency, the DGOA-based approach also demonstrates the fastest convergence rate, requiring only 27 epochs on average to reach stability—compared to 34 for the recurrent method and over 40 for the other baselines. This 7–15 epoch improvement indicates that DGOA learns optimal hyperparameter configurations significantly faster, owing to its dynamic population control and adaptive swarm interaction mechanism. The earlier stabilization demonstrates that DGOA efficiently balances exploration and exploitation, reducing unnecessary evaluations while adapting to evolving data distributions. This rapid convergence not only accelerates model readiness for real-time deployment but also minimizes computational overhead, underscoring the practical and statistical significance of the proposed algorithm over existing state-of-the-art methods.

Furthermore, the runtime per update (45.5 ms) is the lowest among all compared methods, even outperforming the simpler random-search-based strategy. This indicates that the dynamic population control and reduced redundant evaluations in DGOA translate to tangible computational gains, a critical requirement for real-time industrial analytics where continuous data inflow demands minimal latency.

From a conceptual standpoint, these results validate the core motivation of integrating dynamic swarm intelligence with incremental learning. While Gunasekara et al. (2022) and Liu et al. (2023) effectively address online hyperparameter tuning, their methods rely on static adaptation frequencies or discrete decision updates that may lag behind rapid data stream changes. Similarly, the recurrent approach (Im et al., 2021) achieves fine-grained hyperparameter updates but at the expense of higher computational cost due to continuous gradient tracking. In contrast, the proposed DGOA-based model utilizes distributed swarm interactions and real-time adjustment of the social interaction parameters to achieve both global search efficiency and local refinement simultaneously.

### Experiment 8: benchmark comparison with CEC competition algorithms

To further validate the optimization capability of the proposed DGOA, a comparative study was conducted against four leading CEC competition algorithms: (1) LSHADE (Linear Population Size Reduction SHADE), which integrates differential evolution with linear population decay to improve convergence; (2) LSHADE-CnEpSin, an adaptive variant incorporating control parameter coevolution and sinusoidal parameter adaptation; (3) EBOwithCMAR (Elite-Based Optimization with Covariance Matrix and Archive Retention), a covariance-driven elite learning scheme that enhances exploration-exploitation balance; and (4) SALSHADE (Self-Adaptive LSHADE), which dynamically tunes its scaling and crossover parameters during evolution.

Each algorithm was tested using the CEC 2017 benchmark suite (http://github.com/P-N-Suganthan/CEC2017-BoundContrained) under the same experimental settings: population size = 30, dimensions = 30, maximum function evaluations = 300,000, and 30 independent runs. The mean error, standard deviation, and Wilcoxon significance test were computed for each algorithm across all 30 functions. This evaluation complements the industrial use-case experiments by assessing DGOA’s global optimization reliability on standardized test functions.

**Table 2:** Comparison of the proposed DGOA with recent CEC competition algorithms (CEC 2017, 30D).

Algorithm	Mean error (↓)	Std. Dev.	Rank (Avg.)	Key mechanism
LSHADE	1.72 × 10^−2^	2.11 × 10^−3^	3.4	Linear population size reduction with DE mutation
LSHADE-CnEpSin	1.25 × 10^−2^	1.93 × 10^−3^	2.9	Parameter coevolution and sinusoidal adaptation
EBOwithCMAR	1.14 × 10^−2^	1.78 × 10^−3^	2.6	Elite-based covariance learning with adaptive archive
SALSHADE	1.08 × 10^−2^	1.69 × 10^−3^	2.3	Self-adaptive scaling and crossover control
**Proposed DGOA**	**8.6 × 10** ^**−3**^	**1.25 × 10** ^**−3**^	**1.8**	Dynamic step-size control and adaptive swarm interaction

The results in Table 2 demonstrate that the proposed DGOA achieves the lowest mean error (8.6 × 10^−3^) among all compared algorithms, indicating superior convergence accuracy and solution quality across the 30 CEC 2017 benchmark functions. A lower mean error reflects the algorithm’s enhanced ability to approach the true global optimum, thereby confirming its strong optimization precision. Moreover, DGOA exhibits the smallest standard deviation (1.25 × 10^−3^), evidencing high robustness and consistent performance over 30 independent runs. This stability suggests that the algorithm effectively mitigates the influence of random initialization and stochastic search variations. In comparison, competing methods such as LSHADE and LSHADE-CnEpSin show higher mean errors and larger deviations, indicating greater variability and less reliable convergence behavior under identical conditions.

The lowest average rank (1.8) attained by DGOA further substantiates its overall superiority, showing that it consistently outperforms the benchmark algorithms across different function categories (unimodal, multimodal, hybrid, and composite). This advantage stems primarily from DGOA’s dynamic step-size control and adaptive swarm interaction mechanisms, which facilitate a balanced trade-off between global exploration and local exploitation. These adaptive features enable the algorithm to maintain population diversity in early stages and intensify local search near promising regions during later iterations, thereby avoiding premature convergence and accelerating toward optimal solutions. Compared with the adaptive parameter coevolution in LSHADE variants and the covariance-based elite learning in EBOwithCMAR, DGOA demonstrates higher convergence reliability, adaptability, and computational efficiency.

The Wilcoxon rank-sum significance test further supports the observed performance trends, revealing that DGOA’s improvements over competing algorithms are statistically significant at the 5% confidence level ($\:p\:<\:0.05$) for the majority of the benchmark functions. This result confirms that the performance gains are not due to random fluctuations but reflect genuine methodological advantages. Consequently, the combination of superior accuracy, stability, and statistically validated performance demonstrates that DGOA provides a reliable and effective optimization framework, outperforming leading CEC competition algorithms under equivalent experimental conditions.

### Experiment 9: comparison with state-of-the-art drift-aware data stream classifiers

The objective of this experiment is to comprehensively evaluate the effectiveness of the proposed DGOA-based incremental learning framework in comparison with recent state-of-the-art data stream classifiers that explicitly address concept drift through neural or ensemble-based adaptation mechanisms. To this end, three representative methods were selected, each embodying a distinct strategy for handling non-stationary data streams. DeepStreamEnsemble^69^ integrates deep neural networks with explicit drift detection by monitoring hidden-layer activations and prediction stability, enabling rapid adaptation through a streaming classifier ensemble without full retraining. The Adaptive Tree-Like Neural Network (ATNN)^70^ addresses catastrophic forgetting by dynamically expanding its neural architecture; when concept drift is detected via classification error patterns, new branches are grown to capture emerging concepts while preserving previously learned knowledge. Broad Ensemble Learning System (BELS)^71^, in contrast, adopts an ensemble-based incremental learning paradigm by extending the Broad Learning System to streaming environments, updating multiple lightweight learners using mini-chunks to balance stability and adaptability. For all methods, the default configurations recommended in their respective publications were adopted, with only minor adjustments to accommodate the scale and temporal resolution of the electricity market dataset. Specifically, DeepStreamEnsemble employs a lightweight deep neural backbone with its original drift detection mechanism (DSE-DD), ATNN initializes with a shallow tree structure and predefined branch-growth thresholds, and BELS is implemented using an ensemble of 10 base learners updated over fixed-size mini-chunks of 500 samples.

To ensure a fair and consistent evaluation, all methods were tested under the same streaming protocol and data preprocessing pipeline used in Experiments 1 and 2. The Australian electricity market dataset, consisting of 45,312 time-ordered samples labeled as UP or DOWN, was processed sequentially to simulate real-time data arrival, with model updates performed at each window. Performance was assessed using three complementary metrics: classification accuracy, which measures the overall correctness of predictions; macro-average F1 score, which evaluates balanced predictive performance across both classes under potential class imbalance; and update latency, which quantifies the time required to incorporate new data into the model, reflecting real-time responsiveness. Each experiment was repeated 10 independent times, and results are reported as mean ± standard deviation to account for stochastic variability.

Table 3 presents a comprehensive comparison of the proposed DGOA-Optimized MLP against baseline and state-of-the-art data stream classifiers under non-stationary (drifting) data conditions. The Baseline MLP, which lacks any drift-handling or adaptive mechanism, achieves the lowest performance with an accuracy of 83.4 ± 0.41% and a macro-F1 score of 0.87 ± 0.02, indicating its limited ability to cope with evolving data distributions. Its relatively higher update latency (0.25 s) further reflects inefficiencies caused by frequent weight adjustments without informed adaptation. In contrast, the DGOA-Optimized MLP attains the highest overall predictive performance, achieving 88.7 ± 0.29% accuracy and 0.91 ± 0.01 macro-F1, while simultaneously exhibiting the lowest update latency (0.17 s). These results demonstrate that dynamic optimization of learning hyperparameters enables the model to adapt smoothly and efficiently to concept drift, maintaining both accuracy and computational responsiveness.

**Table 3:** Comparative results.

Method	Accuracy (%)	Macro-F1 (%)	Update Latency (s)
Baseline MLP (no drift adaptation)	83.4 ± 0.41	0.87 ± 0.02	0.25
DGOA-Optimized MLP (ours)	**88.7 ± 0.29**	**0.91 ± 0.01**	0.17
DeepStreamEnsemble^69^	87.9 ± 0.33	0.90 ± 0.02	0.45
ATNN (Adaptive Tree-like NN)^70^	86.8 ± 0.37	0.89 ± 0.02	0.29
BELS (Broad Ensemble)^71^	87.2 ± 0.35	0.90 ± 0.02	0.23

Among the state-of-the-art drift-aware methods, DeepStreamEnsemble performs competitively with 87.9 ± 0.33% accuracy and 0.90 ± 0.02 macro-F1, confirming the effectiveness of explicit drift detection based on neural activations. However, this performance comes at a significant computational cost, as reflected by the highest update latency (0.45 s), due to ensemble maintenance and drift detection overhead. ATNN achieves 86.8 ± 0.37% accuracy and 0.89 ± 0.02 macro-F1, indicating that its structural growth strategy can capture new concepts, but its branch-expansion mechanism introduces adaptation delays under frequent or gradual drift. BELS, with 87.2 ± 0.35% accuracy and 0.90 ± 0.02 macro-F1, offers a balanced trade-off between performance and efficiency, yet its reliance on mini-chunk updates limits responsiveness to rapid distributional changes.

From a metric-level perspective, accuracy reflects overall correctness and shows that the proposed model improves absolute predictive performance by 5.3% points over the non-adaptive baseline and by 0.8–1.9 points over competing drift-aware models. Macro-F1, which equally weights both classes, confirms that these gains are not driven by class imbalance but by consistently improved decision boundaries under drift. The higher macro-F1 of 0.91 achieved by the proposed model indicates superior robustness to evolving class distributions. Update latency, a critical metric for streaming applications, highlights a key advantage of the proposed approach: unlike ensemble-based or structure-adaptive methods, the DGOA-Optimized MLP embeds adaptation directly within the learning process, avoiding costly drift detection or model expansion steps.

Overall, these results demonstrate that the proposed method is particularly well-suited for non-stationary data streams, where concept drift is continuous rather than abrupt. By jointly optimizing network weights and learning hyperparameters online, the DGOA-Optimized MLP achieves faster, smoother adaptation compared to methods that rely on discrete drift detection or structural modifications. This fundamental difference in adaptation strategy explains why the proposed model consistently delivers higher accuracy, better class-balanced performance, and lower latency, making it a more effective and practical solution for real-time streaming environments.

### Experiment 10: ablation study (evaluating the contribution of grasshopper optimization in non-stationary data streams)

The primary objective of this ablation study is to quantify the specific contribution of the Grasshopper Optimization component to the performance of the proposed incremental neural network in streaming environments. To achieve this, we compare the DGOA-Optimized MLP, which employs the Grasshopper-inspired dynamic hyperparameter adaptation, against a non-swarm variant that relies on a standard incremental search approach. This comparison allows us to assess three key aspects: the effectiveness in improving classification accuracy and macro-F1 score under both stationary and non-stationary (concept-drifting) data streams; the efficiency of update latency, highlighting how dynamic exploration-exploitation control can reduce computational overhead while maintaining timely adaptation; and the robustness across repeated runs, demonstrating stable convergence and low variance due to the Grasshopper-inspired step-size adjustments and social interaction mechanisms.

For the non-swarm baseline, the IRS-Optimized MLP^72^ utilizes an incremental random search (IRS) to tune key hyperparameters, specifically the learning rate and momentum, within predefined ranges. Unlike DGOA, IRS randomly samples candidate hyperparameter values at each update without leveraging any guided exploration or population-based interactions. This method provides a fair comparison by maintaining identical base model architecture—a single-layer MLP with 20 hidden neurons—and the same streaming protocol, dataset, and evaluation metrics. Both models are incrementally updated on 500-sample windows from the Australian electricity market dataset (45,312 samples, UP/DOWN labels), and each experiment is repeated 10 times to report mean and standard deviation for classification accuracy (%), macro-average F1 score, and update latency (s), ensuring consistency and comparability across methods.

**Table 4:** Ablation results.

Method	Accuracy (%)	Macro-F1 (%)	Update Latency (s)
IRS-Optimized MLP (non-GOA)	86.3 ± 0.38	0.89 ± 0.02	0.21
**DGOA-Optimized MLP (ours)**	**88.7 ± 0.29**	**0.91 ± 0.01**	**0.17**

The results in Table 4 clearly highlight the contribution of the Grasshopper Optimization component (DGOA) to the performance of the proposed incremental MLP model. The DGOA-Optimized MLP achieves an accuracy of 88.7% ± 0.29, which is 2.4% higher than the IRS-Optimized MLP (non-GOA), which records 86.3% ± 0.38. This improvement demonstrates that the dynamic exploration–exploitation mechanism of the Grasshopper optimizer effectively identifies better hyperparameter configurations (learning rate and momentum) in streaming environments, even under concept drift conditions. Similarly, the macro-F1 score improves from 0.89 ± 0.02 (IRS) to 0.91 ± 0.01 (DGOA), indicating more balanced precision and recall across the UP and DOWN classes, which is particularly important given the class imbalance in the dataset (19,237 UP vs. 26,075 DOWN samples). These results show that DGOA not only improves overall predictive accuracy but also stabilizes class-level performance, reducing the risk of bias toward the majority class.

In terms of computational efficiency, the update latency metric reflects the time taken for the model to incrementally update per streaming window. The IRS-Optimized MLP exhibits an update latency of 0.21 s, whereas the DGOA-Optimized MLP reduces this latency to 0.17 s, representing a ~ 19% reduction in update time. This difference is attributed to DGOA’s adaptive step-size mechanism, which enables faster convergence to optimal hyperparameters within each stream window without requiring exhaustive or repeated evaluations. The lower latency indicates that the DGOA-based model is better suited for real-time streaming applications, maintaining high accuracy while efficiently handling new incoming data, which is critical for industrial or high-frequency decision-making scenarios.

Despite the relatively small absolute increase in accuracy (2.4%), this improvement is practically significant in the context of non-stationary data streams, where even minor gains reflect better adaptation to concept drift and robustness across sequential updates. Compared to the IRS baseline, which relies on random hyperparameter sampling, the DGOA component provides structured, guided exploration of the search space, avoiding suboptimal configurations and reducing variance across runs, as reflected by the smaller standard deviation (± 0.29 vs. ±0.38). Overall, the DGOA-Optimized MLP demonstrates clear advantages: higher accuracy, more balanced class performance (macro-F1), and faster incremental updates, confirming that the Grasshopper-inspired hyperparameter optimization is a key factor in achieving stable, efficient, and adaptive performance in streaming neural networks.

### Computational complexity analysis

The computational complexity of the proposed DGOA can be analyzed in terms of the number of search agents ($\:N$), maximum iterations ($\:T$), and the dimension of the search space ($\:D$) corresponding to the optimized parameters (here, learning rate $\:\eta\:$ and momentum $\:\mu\:$). For each iteration, the algorithm performs three primary operations: (1) Fitness evaluation for each agent, which requires training the MLP for a few epochs ($\:O\left(E\right)$, $\:E$ represents the number of training epochs; (2) Force and position updates for each agent ($\:O\left(ND\right)$; (3) Dynamic parameter updates for the step size ($\:{\varDelta\:}^{\left(t\right)}$) and attraction coefficient ($\:{c}_{t}$) ($\:O\left(N\right)$. Hence, the total computational complexity of DGOA per iteration is $\:O(T\times\:(N\times\:(D+E)\left)\right)$. Since $\:D$ and $\:E$ are small constants in this application ($\:D=2,\:E\approx\:few\:epochs$), the complexity simplifies to $\:O\left(TN\right)$, similar to the standard GOA.

However, DGOA’s adaptive control strategy accelerates convergence by reducing the number of required iterations ($\:{T}_{DGOA}<{T}_{GOA})$. Therefore, while per-iteration cost is slightly higher due to dynamic coefficient updates, the overall computational cost remains comparable or even lower than GOA when measured by total runtime to convergence. Empirical runtime measurements confirmed this efficiency: DGOA achieved convergence in approximately 25 epochs, compared to 40–45 epochs for the baseline GOA, representing an average 35–40% reduction in training time. This efficiency gain makes DGOA suitable for real-time and resource-constrained industrial data stream applications.

**Table 5:** Comparative computational complexity and runtime efficiency between GOA and DGOA.

Algorithm	Per-iteration time complexity	Space complexity	Avg. iterations to converge	Relative runtime	Remarks
GOA	$\:O\left(TN\right)$	$\:O\left(ND\right)$	40–45	100%	Fixed coefficients, slower convergence
DGOA	$\:O\left(TN\right)\:$(slightly higher constant)	$\:O\left(ND\right)$	~ 25	~ 60–65%	Dynamic step size and attraction improve convergence

As shown in Table 5, both GOA and DGOA share the same asymptotic time complexity of $\:\boldsymbol{O}\left(\boldsymbol{T}\boldsymbol{N}\right)$, where $\:N$ denotes the number of search agents and $\:T$ the number of iterations. This is because both algorithms rely on identical population-based position and force update mechanisms, and the dimensionality of the search space remains small ($\:D=2$) in this application. The proposed DGOA introduces adaptive updates for the step size and attraction coefficient, which slightly increases the constant factor of the per-iteration cost; however, this does not alter the overall asymptotic complexity.

In terms of space complexity, both algorithms require $\:O\left(ND\right)$ memory to store agent positions and associated parameters. Since DGOA does not introduce additional population-level data structures, its memory requirements remain comparable to those of the standard GOA.

Importantly, the key advantage of DGOA lies in its faster convergence behavior. Due to its dynamic control strategy, DGOA converges in approximately 25 iterations, compared to 40–45 iterations for GOA. This reduction of nearly 35–40% in the number of required iterations leads to a significantly lower total runtime to convergence, despite the slightly higher per-iteration overhead. Empirical runtime measurements confirm that DGOA achieves only 60–65% of the runtime required by GOA. These results demonstrate that the proposed DGOA offers superior computational efficiency in practice, making it particularly suitable for real-time and resource-constrained industrial data stream applications.

### Rationale and problem mitigation analysis of the proposed algorithm

Traditional online and incremental learning approaches for hyperparameter optimization—such as adaptive random search^66^, bandit-based decision frameworks^67^, and gradient-based recurrent learning^68^—face three key challenges:

- Limited adaptability to rapid distributional shifts in streaming data,
- Premature convergence caused by fixed or poorly balanced exploration–exploitation dynamics,
- High computational cost due to frequent retraining or continuous gradient tracking.

The proposed DGOA–based incremental neural network model is specifically designed to address these issues. Unlike static swarm optimizers, DGOA employs a dynamic population control mechanism that adaptively tunes the social interaction coefficients of the grasshopper swarm according to the degree of non-stationarity in the incoming data stream. When the data variance increases, the exploration radius is expanded to capture new patterns; conversely, during stable phases, the algorithm shifts to localized exploitation for fine-grained hyperparameter refinement. This self-regulating adaptive behavior enables DGOA to maintain robustness and prevent premature stagnation even under evolving industrial data environments.

 In addition, DGOA eliminates the need for full model retraining by performing incremental hyperparameter updates synchronized with the streaming data window, thereby significantly reducing computational overhead. The empirical results presented in Table 8 validate this design rationale: DGOA achieves the highest accuracy (95.62%) and macro-F1 score (0.947), while converging faster (27 epochs) and consuming less runtime per update (45.5 ms) compared to the benchmark algorithms. These outcomes demonstrate that the proposed DGOA framework effectively balances adaptability, efficiency, and accuracy—mitigating the key deficiencies of existing online optimization methods—and thereby represents a scalable and intelligent solution for real-time industrial big data stream classification^73,74^.

## Conclusion

This research presents an incremental learning model for optimizing the hyperparameters of MLP neural networks, employing the DGOA for industrial big data streams. The approach addresses the challenges of dynamic data, concept drift, and the high computational cost of retraining traditional neural networks. By combining incremental learning with DGOA, the framework continuously tunes critical hyperparameters—such as learning rate and momentum—adapting in real time to changes in incoming data streams.

The proposed model introduces several key contributions. First, it provides an online hyperparameter optimization mechanism, reducing reliance on static retraining approaches. Second, the integration of swarm intelligence enables efficient exploration of the hyperparameter search space, achieving faster convergence and higher accuracy than traditional methods. Third, the incremental learning strategy allows the model to maintain high performance while minimizing computational overhead, demonstrating adaptability to both stationary and non-stationary data streams.

Experimental results validate the effectiveness of the proposed approach. Across use cases including predictive maintenance, anomaly detection, and fraud detection, the DGOA-optimized MLP consistently outperformed baseline and batch-learning models. The framework achieved high classification accuracy (up to 96.5%), low latency in incremental updates (0.15–0.17 s), and robust adaptation to concept drift, confirming both scalability and generalization. Hyperparameter sensitivity analysis further supports the stability of the chosen configuration (learning rate = 0.01, momentum = 0.8, 5 hidden layers, batch size 64).

Despite these strengths, the proposed model has limitations. Its scalability to extremely large or highly complex datasets may require further enhancement. The adaptability of DGOA in highly dynamic, noisy, or heterogeneous industrial environments has not been fully explored. Additionally, the current framework focuses primarily on MLP networks, and its performance on other neural architectures remains to be evaluated.

Future research could focus on enhancing the robustness and scalability of DGOA in extreme conditions and highly dynamic environments. Integration with transfer learning or hybrid approaches could further improve adaptability and performance across diverse industrial domains. Extending the framework to support other neural network architectures, heterogeneous data types, and real-time multi-modal streaming data will broaden its applicability.

While the proposed framework effectively addresses virtual drift and real concept drift through online detection and adaptive hyperparameter optimization, it does not explicitly handle more complex drift types such as recurring drift, feature-space evolution, or label-space expansion. In future work, the framework will be extended by integrating memory-based drift recall mechanisms for recurring concepts, adaptive feature selection to handle evolving feature spaces, and neural architecture search (NAS) to dynamically adjust network structure in response to structural drift. These enhancements will further improve the robustness of the model in highly complex and long-term streaming environments.

## Supplementary Information

Below is the link to the electronic supplementary material.

Supplementary Material 1

## Data Availability

The datasets analyzed during the current study are publicly available and can be accessed from the following sources: 1. NSW Australia Electricity Demand (2018–2023) – Kaggle repository: (https:/www.kaggle.com/datasets/joebeachcapital/nsw-australia-electricity-demand-2018-2023). 2. NASA C-MAPSS (Jet Engine Simulated Data) – Kaggle repository (originally from NASA Prognostics Data Repository): (https:/www.kaggle.com/datasets/palbha/cmapss-jet-engine-simulated-data) Original source: NASA Prognostics Data Repository. 3. SWaT (Secure Water Treatment) Dataset – iTrust Centre for Research in Cyber Security: (https:/itrust.sutd.edu.sg/itrust-labs_datasets/dataset_info). 4. IEEE-CIS Fraud Detection Dataset – Kaggle competition repository: (https:/www.kaggle.com/competitions/ieee-fraud-detection). All datasets are publicly available, and we confirm that their use complies with the licensing and access conditions specified by each repository and original data provider.
