# Supplementary material for: Intelligent incremental classification using a dynamic grasshopper-enhanced neural network for data streams
Source: Sci Rep. 2026 Feb 26;16:7730. doi: 10.1038/s41598-026-38571-y (PMC12949023; doi:10.1038/s41598-026-38571-y)
Supplement: Supplementary file 1 — Supplementary Material 1 [file 41598_2026_38571_MOESM1_ESM.docx]

# DGOA-MLP Streaming Implementation

This supplementary document contains a complete, self-contained Python implementation of the proposed Dynamic Grasshopper Optimization Algorithm (DGOA) for hyperparameter tuning of an incremental MLP classifier in a streaming (train-and-forget) setting. The code is designed for reproducibility and is provided as a single Python module.

"""
DGOA-MLP Streaming Classifier (Train-and-Forget)
------------------------------------------------

This file implements a reproducible, self-contained reference implementation
of the proposed Dynamic Grasshopper Optimization Algorithm (DGOA) for
hyperparameter tuning of an incremental MLP classifier in a streaming setting.

The implementation is intentionally simplified and modular, suitable for
research reproduction and extension.

Dependencies:
- numpy
- scikit-learn
- pandas (optional, for dataset handling)
- tqdm (optional, for progress bars)

Usage:
- Define a streaming dataset generator (windowed).
- Instantiate StreamingDGOA_MLP.
- Call fit_stream(stream_generator).

from dataclasses import dataclass
from typing import Callable, Iterable, Optional, Tuple, Dict, Any, List
import numpy as np
from sklearn.neural_network import MLPClassifier
from sklearn.preprocessing import MinMaxScaler
from sklearn.metrics import accuracy_score, precision_score, recall_score, f1_score
from sklearn.base import clone
from tqdm import tqdm


# ----------------------------
# Utility functions
# ----------------------------

def clip(x: np.ndarray, lo: float, hi: float) -> np.ndarray:
 return np.minimum(np.maximum(x, lo), hi)


def normalize_window(X: np.ndarray) -> np.ndarray:
 """
 Min-max normalization per feature for a window.
 """
 scaler = MinMaxScaler()
 return scaler.fit_transform(X)


def window_stream(X: np.ndarray, y: np.ndarray, window_size: int, step: int = None):
 """
 Generator yielding sliding windows (X_w, y_w).
 """
 if step is None:
 step = window_size
 n = X.shape[0]
 for start in range(0, n, step):
 end = min(start + window_size, n)
 if end - start < 1:
 break
 yield X[start:end], y[start:end]


# ----------------------------
# DGOA components
# ----------------------------

@dataclass
class Grasshopper:
 eta: float
 mu: float
 delta: float
 fitness: float = np.inf


@dataclass
class DGOAConfig:
 eta_min: float = 1e-4
 eta_max: float = 1e-1
 mu_min: float = 0.0
 mu_max: float = 0.99
 delta_min: float = 0.01
 delta_max: float = 0.5
 c_min: float = 0.0001
 c_max: float = 1.0
 pop_size: int = 20
 max_iter: int = 30
 fitness_metric: str = "loss" # "loss" or "accuracy"
 random_state: int = 42


class DGOAOptimizer:
 """
 Dynamic Grasshopper Optimization Algorithm for optimizing (eta, mu).
 """

 def __init__(self, config: DGOAConfig):
 self.cfg = config
 self.rng = np.random.default_rng(config.random_state)

 def _step_size(self, t: int) -> float:
 return self.cfg.delta_min + (t / self.cfg.max_iter) * (self.cfg.delta_max - self.cfg.delta_min)

 def _attraction_coef(self, t: int) -> float:
 return self.cfg.c_max - (t / self.cfg.max_iter) * (self.cfg.c_max - self.cfg.c_min)

 def _scaling_function(self, r: float) -> float:
 # Standard GOA scaling function
 f = 0.5 # attraction strength
 l = 1.5 # attraction length scale
 return f * np.exp(-r / l) - np.exp(-r)

 def _interaction_force(self, positions: np.ndarray, i: int) -> np.ndarray:
 """
 Compute the social interaction force F_i^(t) for agent i.
 """
 xi = positions[i]
 force = np.zeros_like(xi)
 for j in range(len(positions)):
 if j == i:
 continue
 xj = positions[j]
 dist = np.linalg.norm(xj - xi)
 if dist == 0:
 continue
 s = self._scaling_function(dist)
 force += s * (xj - xi) / dist
 return force

 def optimize(self,
 fitness_fn: Callable[[float, float], float],
 bounds: Tuple[Tuple[float, float], Tuple[float, float]]) -> Tuple[float, float, float]:
 """
 Optimize eta and mu using DGOA.
 Returns (eta_best, mu_best, best_fitness).
 """
 eta_bounds, mu_bounds = bounds
 pop: List[Grasshopper] = []
 for _ in range(self.cfg.pop_size):
 eta = self.rng.uniform(*eta_bounds)
 mu = self.rng.uniform(*mu_bounds)
 delta = self.rng.uniform(self.cfg.delta_min, self.cfg.delta_max)
 pop.append(Grasshopper(eta=eta, mu=mu, delta=delta))

 # Evaluate initial fitness
 positions = np.array([[g.eta, g.mu] for g in pop])
 for idx, g in enumerate(pop):
 g.fitness = fitness_fn(g.eta, g.mu)

 best = min(pop, key=lambda g: g.fitness)

 for t in range(1, self.cfg.max_iter + 1):
 delta_t = self._step_size(t)
 c_t = self._attraction_coef(t)

 positions = np.array([[g.eta, g.mu] for g in pop])
 best_pos = np.array([best.eta, best.mu])

 for i, g in enumerate(pop):
 force = self._interaction_force(positions, i)
 xi = np.array([g.eta, g.mu])

 # Position update (Equation 15)
 xi_new = xi + delta_t * (c_t * (best_pos - xi) + force)

 # Clip to bounds
 xi_new[0] = float(clip(xi_new[0], eta_bounds[0], eta_bounds[1]))
 xi_new[1] = float(clip(xi_new[1], mu_bounds[0], mu_bounds[1]))

 g.eta, g.mu = xi_new
 g.fitness = fitness_fn(g.eta, g.mu)

 # update best
 current_best = min(pop, key=lambda g: g.fitness)
 if current_best.fitness < best.fitness:
 best = current_best

 return best.eta, best.mu, best.fitness


# ----------------------------
# Streaming MLP
# ----------------------------

@dataclass
class StreamMetrics:
 accuracy: float
 precision: float
 recall: float
 f1: float


class StreamingDGOA_MLP:
 """
 Streaming classifier using incremental MLP and DGOA hyperparameter optimization.
 """

 def __init__(self,
 mlp_params: Optional[Dict[str, Any]] = None,
 dgoa_cfg: Optional[DGOAConfig] = None,
 window_size: int = 500,
 drift_threshold: float = 0.10,
 reinit_fraction: float = 0.25):
 self.mlp_params = mlp_params or {"hidden_layer_sizes": (50,),
 "activation": "relu",
 "solver": "sgd",
 "batch_size": "auto",
 "max_iter": 1,
 "warm_start": True,
 "random_state": 42}
 self.dgoa_cfg = dgoa_cfg or DGOAConfig()
 self.window_size = window_size
 self.drift_threshold = drift_threshold
 self.reinit_fraction = reinit_fraction

 self.model: Optional[MLPClassifier] = None
 self.metrics_history: List[StreamMetrics] = []
 self._baseline_error: Optional[float] = None

 def _train_mlp(self, X: np.ndarray, y: np.ndarray, eta: float, mu: float) -> float:
 """
 Train MLP for one epoch on window and return validation loss.
 For simplicity, we split the window into train/val 80/20.
 """
 if self.model is None:
 self.model = MLPClassifier(**self.mlp_params)

 # Set dynamic hyperparameters
 self.model.set_params(learning_rate_init=eta, momentum=mu)

 # Split into train/validation within window
 n = len(X)
 split = int(0.8 * n)
 X_train, X_val = X[:split], X[split:]
 y_train, y_val = y[:split], y[split:]

 self.model.fit(X_train, y_train)
 y_pred = self.model.predict(X_val)

 # validation loss as (1 - accuracy) for simplicity
 loss = 1.0 - accuracy_score(y_val, y_pred)
 return loss

 def _fitness_from_window(self, X: np.ndarray, y: np.ndarray) -> Callable[[float, float], float]:
 """
 Returns a fitness function for DGOA based on current window.
 """
 def fitness(eta: float, mu: float) -> float:
 # clone the model to avoid changing the running model during optimization
 saved_model = clone(self.model) if self.model is not None else None

 loss = self._train_mlp(X, y, eta, mu)

 # restore
 if saved_model is not None:
 self.model = saved_model

 return loss

 return fitness

 def _detect_drift(self, y_true: np.ndarray, y_pred: np.ndarray) -> bool:
 err = 1.0 - accuracy_score(y_true, y_pred)
 if self._baseline_error is None:
 self._baseline_error = err
 return False
 if abs(err - self._baseline_error) / max(self._baseline_error, 1e-9) > self.drift_threshold:
 self._baseline_error = err
 return True
 return False

 def fit_stream(self,
 stream: Iterable[Tuple[np.ndarray, np.ndarray]],
 verbose: bool = True) -> List[StreamMetrics]:
 """
 Fit the model on a stream of windows.
 """
 for X_w, y_w in tqdm(stream, disable=not verbose, desc="Streaming windows"):
 X_w = normalize_window(X_w)

 # initial training (first window)
 if self.model is None:
 # use default hyperparameters for initialization
 self.model = MLPClassifier(**self.mlp_params)
 self.model.fit(X_w, y_w)
 continue

 # Evaluate current performance to detect drift
 y_pred = self.model.predict(X_w)
 drift = self._detect_drift(y_w, y_pred)

 if drift:
 # reinitialize part of the population by increasing exploration
 self.dgoa_cfg.delta_max = min(1.0, self.dgoa_cfg.delta_max * 1.5)
 self.dgoa_cfg.c_max = min(1.0, self.dgoa_cfg.c_max * 1.2)

 # DGOA optimization on current window
 optimizer = DGOAOptimizer(self.dgoa_cfg)
 fitness_fn = self._fitness_from_window(X_w, y_w)
 eta_best, mu_best, _ = optimizer.optimize(
 fitness_fn,
 bounds=((self.dgoa_cfg.eta_min, self.dgoa_cfg.eta_max),
 (self.dgoa_cfg.mu_min, self.dgoa_cfg.mu_max))
 )

 # Incremental training with optimized hyperparameters
 self._train_mlp(X_w, y_w, eta_best, mu_best)

 # record metrics on this window
 y_pred2 = self.model.predict(X_w)
 metrics = StreamMetrics(
 accuracy=float(accuracy_score(y_w, y_pred2)),
 precision=float(precision_score(y_w, y_pred2, average="weighted", zero_division=0)),
 recall=float(recall_score(y_w, y_pred2, average="weighted", zero_division=0)),
 f1=float(f1_score(y_w, y_pred2, average="weighted", zero_division=0))
 )
 self.metrics_history.append(metrics)

 return self.metrics_history


# ----------------------------
# Example usage
# ----------------------------

def example_run():
 """
 Example with synthetic data for reproduction.
 Replace with real streaming dataset generator as needed.
 """
 from sklearn.datasets import make_classification

 X, y = make_classification(n_samples=5000, n_features=20, n_classes=2, random_state=42)
 stream = window_stream(X, y, window_size=500, step=500)

 model = StreamingDGOA_MLP(window_size=500)
 metrics = model.fit_stream(stream, verbose=True)

 print("Final window metrics:", metrics[-1])

if __name__ == "__main__":
 example_run()
